# Interfacial Co‐Operativity Enables Ultrafast Charge Transfer Within the Co‐Fe Prussian Blue Analogue|Zno Heterostructure

**DOI:** 10.1002/chem.202501696

**Published:** 2025-08-22

**Authors:** Ratnadip De, Ruby Phul, Marius Hermesdorf, Jyoti Bisht, Annett Gawlik, Martin Oschatz, Ferdi Karadaş, Benjamin Dietzek‐Ivanšić

**Affiliations:** ^1^ Department of Functional Interfaces Leibniz Institute of Photonic Technology Jena Albert‐Einstein‐Strasse 9 Jena 07745 Germany; ^2^ Institute of Physical Chemistry Friedrich Schiller University Jena Helmholtzweg 4 Jena 07743 Germany; ^3^ Department of Chemistry Main Campus Bilkent University Ankara 06800 Turkey; ^4^ Center for Energy and Environmental Chemistry Friedrich Schiller University Jena Philosophenweg 7a Jena 07743 Germany; ^5^ Institute for Technical Chemistry and Environmental Chemistry Friedrich Schiller University Jena Philosophenweg 7a Jena 07743 Germany; ^6^ Helmholtz Institute for Polymers in Energy Applications Jena (HIPOLE Jena) Lessingstraße 12–14 Jena 07743 Germany; ^7^ UNAM–National Nanotechnology Research Center Bilkent University Ankara 06800 Turkey; ^8^ Leibniz Institute of Surface Engineering Permoser Strasse 15 Leipzig 04318 Germany

**Keywords:** interfacial charge transfer, prussian blue heterostructure, TR‐VSFG, ultrafast spectrosocopy, water oxidation

## Abstract

Heterostructures of Cobalt‐Iron (Co‐Fe) Prussian blue analogues (PBA) and inorganic semiconductors are attractive materials for photocatalytic and photoelectrochemical water oxidation. Their efficiency is rooted in the charge transfer (CT) at the PBA|semiconductor interface. The interfacial CT, however, often suffers from sluggish kinetics, optimization of which has been elusive. In this work, we investigate PBA|ZnO heterostructures spectroscopically and show that tuning the interfacial composition of the heterostructure presents a synthetic handle to significantly improve interfacial CT. We employ ultrafast transient absorption (TA) spectroscopy to probe the CT kinetics, while interface‐sensitive vibrational spectroscopy, that is, time‐resolved and in‐situ vibrational sum‐frequency generation (VSFG), sheds light on the molecular response to the CT across the interface. These measurements reveal that cooperative intermolecular interactions at the PBA|ZnO interface are key to achieving efficient CT. Furthermore, we relate the CT observed on ps‐timescales to the functional properties of the PBA|ZnO heterostructure in terms of photocatalytic water oxidation, which increases by about 200% in absolute yield as compared to a heterostructure without interfacial co‐operativity. Thus, this work presents for the first time a molecular picture of a PBA|ZnO interface and offers a novel perspective to optimize the CT dynamics in PBA|semiconductor heterostructures by tuning the interfacial chemical structure of PBA.

## Graphical abstract

This combined spectroscopic study shows that when the interfacial composition of PBA|ZnO heterostructure is engineered via a facile synthetic protocol the interfacial CT is promoted by a cooperative mechanism. In this mechanism, pre‐existing oxidized PBA units at the interface facilitate structural rearrangements within the coordination network, thereby lowering the kinetic barrier of the process and improving its water oxidation efficiency.

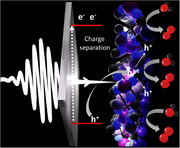

## 1 Introduction

In recent years, Co‐Fe Prussian blue analogues (PBAs)^[^ ^1^, ^2^, ^3^ ^]^ have drawn significant attention as efficient and robust catalysts for water oxidation under neutral and acidic conditions, which is initiated with the activation of water‐coordinated Co^II^ sites upon oxidation to Co^III^.^[^ ^4^, ^5^, ^6^, ^7^ ^]^ One widely explored approach to activate the Co sites in PBA structures is hole transfer from light‐harvesting metal oxide semiconductors such as TiO_2_, BiVO_4,_ Fe_2_O_3_, and SrTiO_3_.^[^ ^8^, ^9^, ^10^, ^11^, ^12^ ^]^ Nonetheless, the light‐activated hole transfer at the PBA|semiconductor interface quite generally suffers from slow reaction kinetics and requires an additional potential bias. For example, a positive bias of ca 0.3 V versus RHE is required for hole transfer from photoexcited BiVO_4_ to PBA.^[^ ^13^ ^]^ Furthermore, such a hole transfer leads to a change in the Co–N bond lengths in the interfacial PBA structure by as large as 0.18 Å.^[^ ^14^, ^15^, ^16^ ^]^ This, in turn, causes a mismatch in unit cell parameters (that requires significant structural rearrangement), which contributes to the sluggish kinetics of this process.^[^ ^14^ ^]^

Key strategies to increase the efficiency of the interfacial charge transfer (CT) dynamics rely on the structural engineering of the PBA‐semiconductor heterostructure to optimize the interfacial interaction. For example, inserting layers of Cu‐Fe Prussian blue between PBA and BiVO_4_ results in an enhanced charge extraction from BiVO_4_ as the extracted charge can be accumulated in the intermediate Cu‐Fe Prussian blue layer.^[^ ^17^ ^]^ Similarly, the presence of phosphate ions between hematite and PBA reduces the CT resistance and suppresses charge recombination at the interface.^[^ ^18^ ^]^ These studies indicate that interfacial engineering is critical in tuning the CT process in PBA‐semiconductor catalysts.

**Figure 1:**
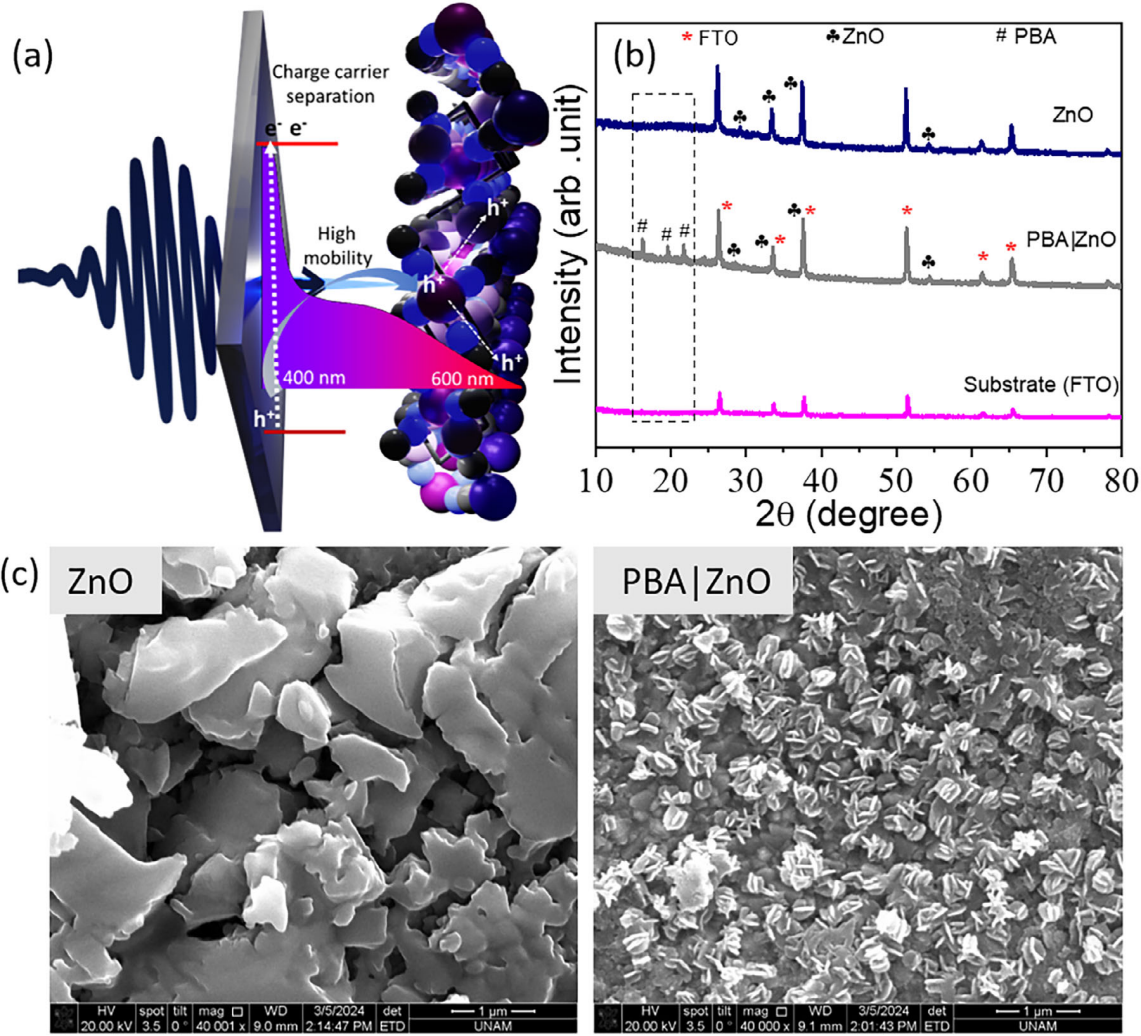
Structure of PBA|ZnO. a) Schematic representation (not crystal structure) of the PBA|ZnO heterostructure and CT process across PBA|ZnO interface, where wide bandgap of the ZnO provides the driving force for charge separation and higher mobility of charge carriers in ZnO facilitates charge transport to PBA molecules at ZnO surface, b) PXRD patterns, and c) SEM images of ZnO substrate and PBA|ZnO heterostructure.

This work focuses on the impact of the interfacial structure on the CT dynamics at the PBA|ZnO interface. The wide bandgap and high electron mobility of ZnO provide a strong driving force for charge carrier separation and facilitate efficient charge transport to the surface.^[^ ^19^, ^20^, ^21^ ^]^ Hence, PBA structures in contact with a ZnO surface constitute a suitable material for photocatalysis owing to their abundant redox active sites.^[^ ^22^ ^]^ Furthermore, our previous work suggests that the interfacial structure of PBA|ZnO can be tuned by varying the fabrication condition.^[^ ^23^ ^]^ Thus, PBA|ZnO presents a potential platform to study the effect of interfacial structure/interaction on the charge dynamics across the interface of such heterostructures. To this end, we employ ultrafast transient absorption (TA) spectroscopy to investigate the CT dynamics at the PBA|ZnO interface. In addition, vibrational sum‐frequency generation (VSFG) spectroscopy elucidates the molecular structural changes associated with the interfacial CT process. The combined spectroscopic approach reveals ultrafast, that is, sub‐picosecond photoinduced CT in the absence of a bias potential. Our study points to a cooperative mechanism, where pre‐existing oxidized PBA units (acting like nucleation sites) facilitate growth of oxidized PBA domains within the coordination network, thereby promoting CT. The spectroscopic insights add to the output of our electrochemical measurements and valence band (VB) X‐ray photoelectron spectroscopy (XPS) that unveils the band alignment of PBA|ZnO and offers a mechanistic interpretation of water oxidation following the photoexcitation of either PBA or ZnO. Thus, the work presented here offers a novel perspective on the interfacial structure and dynamics (please see Figure 1a for a schematic representation) during light‐driven catalysis. We believe this provides a key to optimizing interfacial coupling and catalytic performance in PBA|semiconductor heterostructures.

## 2 Results and Discussion

PBA|ZnO heterostructure is prepared using a temperature‐controlled hydrothermal fabrication method reported earlier by our group (please see the Supporting Information for more details).^[^ ^24^ ^]^ In brief, ZnO substrates were prepared by atomic layer deposition (ALD), where a ZnO layer with a 50 nm thickness was deposited on the glass substrate. Freshly prepared ZnO substrates were then treated with an aqueous solution of Co(NO_3_)_2_·6H_2_O and K_3_[Fe(CN)_6_] for 12–14 hours at 75 °C, unless mentioned otherwise. The resulting PBA|ZnO was characterized by powder X‐ray diffraction (PXRD) and scanning electron microscopy (SEM) (see Figure 1b,c). In the PXRD pattern (Figure 1b), the low‐intensity peaks at 16.2°, 19.5°, and 21.7° confirm the integration of a thin PBA layer on the ZnO substrates.

### 2.1 ZnO‐Centered Excitation: Hole Transfer across the Pba|Zno Interface

**Figure 2:**
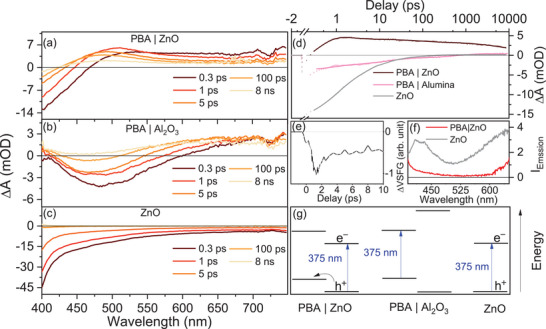
Excitation at 375 nm. Ultrafast TA spectra of a) PBA|ZnO heterostructure, b) PBA immobilized on an electronically inert Al_2_O_3_ surface, and c) bare ZnO substrate upon excitation at 375 nm. d) Temporal evolution of differential absorption at 475 nm for PBA|ZnO, PBA|Al_2_O_3_, and ZnO. Excitation energy was 1 µJ/pulse with a spot diameter of 300 µm, and repetition rate of the laser is 1 kHz. e) TR‐VSFG kinetics of PBA|ZnO under similar excitation conditions. The signal was integrated between 2200 and 2250 cm^− 1^, which is associated with the Co^III^‐CN‐Fe^III^ coordination mode, see Supporting Information for more details concerning TR‐VSFG measurements. f) Emission spectra of bare ZnO and PBA|ZnO, where lowered emission intensity is observed for PBA|ZnO. g) Schematic representation of the physical process–interfacial CT in a sub‐picosecond time scale that causes the appearance of a new absorption band in TA spectrum and modulation of VSFG signal.

Excited‐state photophysics of the PBA|ZnO interface were investigated using TA spectroscopy following the bandgap excitation of ZnO at 375 nm (Figure 2a). It is important to note that multiple photophysical processes can be triggered by such high‐energy excitation (please see Figure S2 for the absorption profile of the samples). For instance, Kamioka et al. pointed out that excitation at 3.1 eV (_∼_400 nm) can induce CN^−^→ Fe^3^⁺ ligand‐to‐metal charge transfer (LMCT) transitions in PBA, which may subsequently lead to intermetallic CT between Co^2^⁺ and Fe^3^⁺.^[^ ^25^ ^]^ Similarly, Zimara et al. reported Co^2^⁺→ Fe^3^⁺ CT in a molecular analogue of this cyanide‐bridged Fe‐Co complex, following excitation at 388 nm.^[^ ^26^ ^]^ Therefore, it is crucial to consider these intermetallic charge dynamics when investigating interfacial CT between PBA and ZnO under similar excitation conditions.

Hence, to isolate the PBA‐centered dynamics, we prepared PBA|Al_2_O_3_ as a control sample. Al_2_O_3_ does not participate in light‐driven redox processes under our experimental conditions and serves solely as a mechanical support for the PBA layer. Thus, upon optical excitation, the TA measurements of PBA|Al_2_O_3_ primarily reveal PBA‐centered dynamics, for example, intermetallic CT. Following 375 nm excitation, the TA spectrum of PBA|Al_2_O_3_ exhibits positive absorption above 600 nm and below 420 nm, along with a ground‐state bleach (GSB) at around 475 nm, as shown in Figure 2b. Kamioka et al. assigned the ESA above 600 nm to CN^−^‐Fe^2^⁺ and Co^3^⁺‐Fe^2^⁺ states, while using excitation conditions comparable to ours.^[^ ^25^ ^]^ Similarly, Zimara et al. observed ESA at 370 nm and 640 nm, which they attributed to the formation of the Co^3^⁺‐Fe^2^⁺ state.^[^ ^26^ ^]^ Hence, in line with previous literature, we assign the spectral features of PBA|Al_2_O_3_ to LMCT (CN^−^‐Fe^2^⁺) and intermetallic CT (Co^3^⁺‐Fe^2^⁺) species following 375 nm excitation. In addition, TA spectra were recorded for bare ZnO. The corresponding data, shown in Figure 2c, reveal a broad negative band that decays within 1 ns.^[^ ^21^ ^]^ This negative transient signal has been previously attributed to the bleach of the valence band (VB) to conduction band (CB) transition in ZnO.^[^ ^21^ ^]^ A very weak, persistent ESA is ascribed to photoinduced absorption of trapped excitons.^[^ ^27^ ^]^

Importantly, the TA spectrum of PBA|ZnO differs significantly from the spectra of its components. Within 1 ps after excitation of the PBA|ZnO composite, a new band emerges at 475–500 nm (Figure 2a), accompanied by a simultaneous decrease in absorption above 600 nm (Figure S5). Also, a concurrent recovery of ground state bleach at 405 nm (bleach of VB→CB transition of ZnO) is observed at the same time scale, which is faster than that of bare ZnO (please see Figures S6,S7). The formation of the new band at 475–500 nm, coupled with the reduction of absorption above 600 nm is unique to PBA|ZnO and thus indicates a dynamic process specific to the PBA|ZnO interface. We tentatively assign this new band to the absorption of oxidized PBA species, formed upon reductive quenching of photoexcited ZnO through hole transfer from the VB of ZnO to the PBA layer. This assignment is consistent with the work of Kulesza et al. and Tacconi et al., who reported increased absorption at ca. 500 nm and 465 nm, respectively, upon electrochemical oxidation of PBA.^[^ ^28^, ^29^ ^]^ Our UV‐Vis spectroelectrochemistry experiments further support this assignment, as the spectrum of electrochemically oxidized PBA closely resembles the spectral profile of the band observed in the TA measurements (see Figure S4).

To gain insights into the molecular response of the PBA interfacial layer upon light‐driven CT, we employed time‐resolved VSFG spectroscopy (TR‐VSFG) (see Supporting Information for experimental details). For PBA|ZnO, the TR‐VSFG signal (e.g., integrated between 2200 and 2250 cm^−1^, see Supporting Information and Figures S10, S11 for details) exhibits the formation of a negative transient signal within approximately 1 ps following 375 nm excitation. Thus, the timescale on which the TR‐VSFG signal builds up is consistent with the timescale previously associated with interfacial CT (Figure 2d–e). In contrast, TR‐VSFG measurements on bare ZnO substrates reveal the ultrafast formation of a positive transient signal, which we tentatively attribute to the absorption of surface‐trapped excitons. The distinct TR‐VSFG signals from the ZnO surface and PBA|ZnO interface indicate different underlying physical processes governing the TR‐VSFG dynamics in these systems. Previous studies by Xiong et al. reported the formation of similar transient negative VSFG signals at a poly(3‐hexylthiophene‐2,5‐diyl)|poly(benzimidazo‐benzophenanthrolin) interface.^[^ ^30^ ^]^ The authors attributed this transient signal to the generation of an electric field at the interface by separated electrons and holes following photoinduced CT.^[^ ^30^ ^]^ This electric field induces additional third‐order nonlinear signals and modulates the VSFG signal, an effect known as the electric‐field‐induced (EFI) effect.^[^ ^31^, ^32^ ^]^ Thus, the transient VSFG signal from the PBA|ZnO interface provides strong evidence for interfacial CT. The observation that the interaction of the 375 nm pump pulse with PBA|ZnO leads to the formation of a TR‐VSFG signal suggests that the photoexcitation results in the separation of charge carriers across the interface of the heterostructure that ultimately led to the oxidation of PBA (transfer of holes from ZnO to PBA). In line with TA and TR‐VSFG measurements, the emission from the PBA|ZnO heterostructure appears quenched when compared to the emission of ZnO alone (Figure 2f). This indicates that light‐induced hole transfer from ZnO to PBA competes with the radiative recombination of ZnO.^[^ ^33^ ^]^

### 2.2 Unusually Fast Interfacial CT Upon Excitation of ZnO

The ultrafast light‐induced CT at the PBA|ZnO interface is particularly noteworthy, as hole transfer at other PBA|semiconductor interfaces, such as BiVO_4_|CoFe PBA, typically occurs on a microsecond timescale and requires a positive electronic bias. Sato et al. highlighted that PBA‐centered intermetallic CT, involving Co^II^→ Co^III^ oxidation, induces significant changes in Co–N bond lengths (up to 0.18 Å), contributing to the barrier for CT.^[^ ^14^ ^]^ Further, Cammarata et al. reported that such intermetallic CT (in aqueous solution, intermetallic CT happens in 200 fs) follows the spin transition at the Co center after excitation and invloves structural distortions like lattice bending and expansion within the CoFe PBA framework.^[^ ^16^ ^]^ However, it is important to note here that when CoFe PBA structures were grown within Nafion films, the formation of CT (intermetallic) states occurred over 25 ps (in comparison to 200 fs in solution), following local lattice distortions, such as the reduction of Co–Fe bond distances post‐excitation.^[^ ^25^ ^]^ Such a striking difference in the time scale demonstrates that structural reorganizations associated with CT can be particularly challenging in rigid environments (as in PBA|ZnO).

Nevertheless, the observation of sub‐picosecond hole transfer in the PBA|ZnO heterostructure suggests that these barriers are significantly reduced compared to other PBA|semiconductor interfaces. To investigate the origins of this enhancement in CT efficiency, we explored the role of interfacial structure in PBA|ZnO.

For comparison, we prepared a control sample, PBA|ZnO‐RT, known to have a distinct interfacial structure from PBA|ZnO based on our previous work. Previously we showed that the PBA|ZnO interface contains oxidized PBA motifs (e.g., Fe^III^–CN–Co^III^ and/or Fe^III^–NC–Co^III^), whereas PBA|ZnO‐RT, prepared at room temperature, predominantly features Fe^II^–CN–Co^II^, Fe^II^–CN–Co^III^, and Fe^III^–CN–Co^II^ coordination motifs.^[^ ^23^ ^]^ Comparative XPS analysis of PBA|ZnO and PBA|ZnO‐RT (see Figure S19 and Table S1 in the Supporting Information) also supports this distinction.

**Figure 3:**
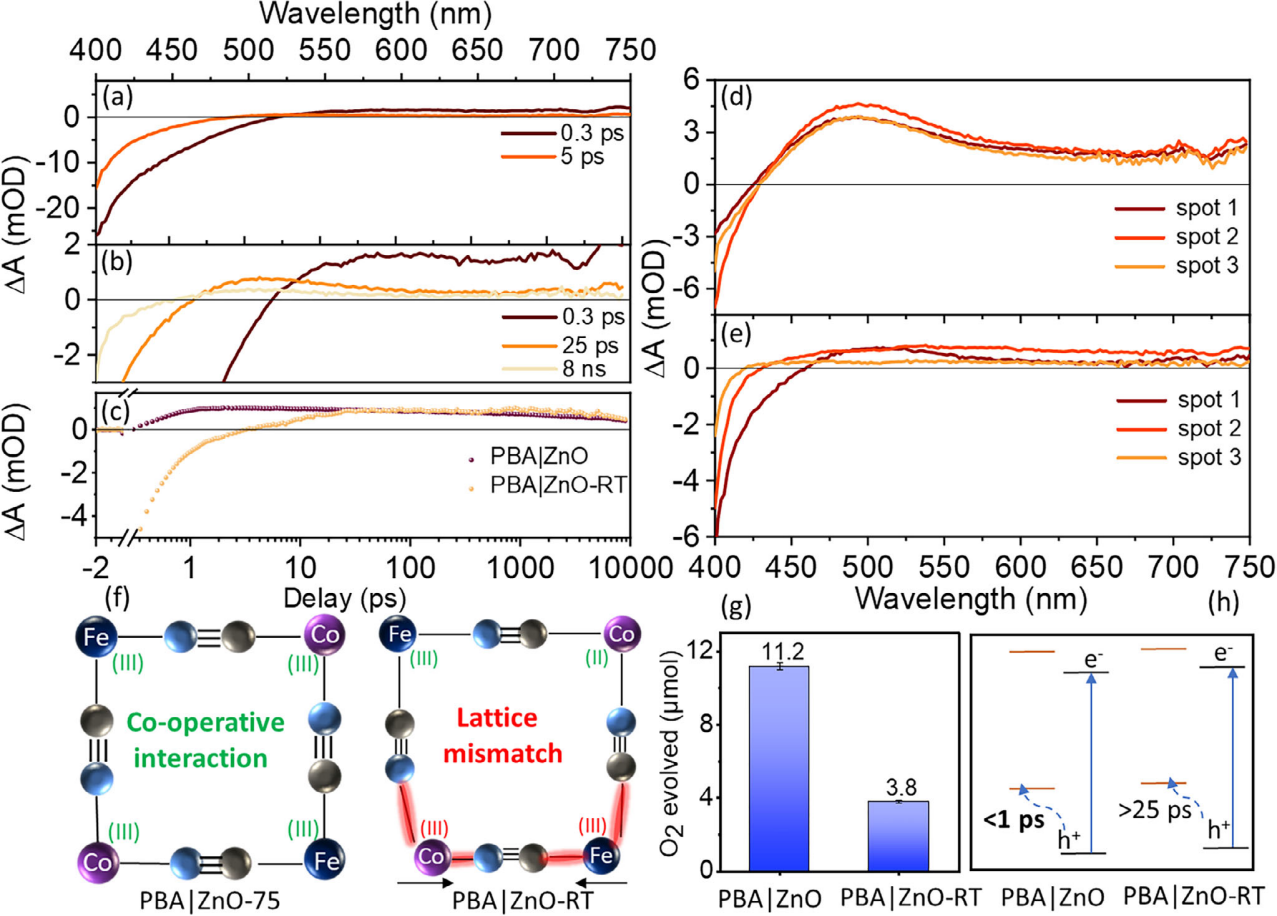
The origin of ultrafast CT. a)‐b) TA spectra of PBA|ZnO‐RT heterostructure with excitation at 375 nm. Excitation energy was 1 µJ/pulse with a spot diameter of 300 µm. c) Comparison of the TA kinetics at 475 nm between PBA|ZnO and PBA|ZnO‐RT, showing different rates of hole transfer in these two samples d)‐e) Representative TA spectra of PBA|ZnO and PBA|ZnO‐RT at 100 ps delay measured on different spots of samples f) A schematic representation of unit cell mismatch due to the change in bond length in PBA|ZnO‐RT, where oxidized PBA motifs are surrounded by unoxidized PBA; on the other hand, PBA|ZnO benefits from the co‐operative interaction between newly oxidized and pre‐oxidized PBA moieties g) The relative yield of photocatalytic water oxidation with PBA|ZnO and PBA|ZnO‐RT samples. For the photocatalytic experiments, the PBA‐coated ZnO substrates were used as a catalyst and a solar simulator was used as a light source (please see the experimental section in Supporting Information for more details). Under the same experimental condition, the PBA deposited on a glass substrate did not show any catalytic activity. h) Schematic of the CT process of two samples.

TA measurements on PBA|ZnO‐RT under conditions identical to those for PBA|ZnO revealed significant differences. PBA|ZnO‐RT exhibits a pronounced GSB but only a weak, broad, and unstructured excited‐state absorption (ESA) at probe wavelengths >525 nm (Figure 3a). A weak band resembling the spectrum of oxidized PBA emerges between 475 and 500 nm after ∼25 ps (Figure 3b). A comparative kinetic analysis at 475 nm and 405 nm, as shown in Figure 3c and S8, highlights the different oxidation/hole transfer rates in PBA|ZnO and PBA|ZnO‐RT (also we note that the slower CT rate of PBA|ZnO‐RT does not improve at higher pump fluence, as shown in Figure S9). This difference points toward the critical role of the interfacial composition of PBA|ZnO on its interfacial CT. It suggests that ultrafast CT happens only when pre‐oxidized PBA is present at the PBA|ZnO interface. Following the arguments by Sato et al., we rationalize this observation in terms of intermolecular co‐operativity.^[^ ^14^ ^]^ Sato et al. pointed out that structural distortion associated with CT can be avoided when species with similar electronic configurations surround the newly generated species (after CT).^[^ ^14^ ^]^ In the case of PBA|ZnO, this is possible since the oxidized PBA motifs after the CT can be surrounded by pre‐oxidized PBA (i.e population of PBA oxidized during fabrication at high temepratures) moieties. This cooperative interaction is absent in PBA|ZnO‐RT, which lacks pre‐oxidized motifs at its interface. Previously, our VSFG spectroelectrochemistry measurements also showed that oxidized PBA moieties act as nucleation sites and favor further growth of the oxidized PBA domain under oxidative conditions.^[^ ^23^ ^]^ Herein, a comparison of the CT dynamics between PBA|ZnO and PBA|ZnO‐RT, alongside insights from our VSFG spectroelectrochemistry results, suggests that intermolecular cooperative interactions in PBA|ZnO play a key role in enabling ultrafast interfacial CT.

Furthermore, measurements at different spots (200–250 µm diameter) on PBA|ZnO‐RT show significant variations in spectral shape and intensity (Figure 3e). In contrast, PBA|ZnO displays consistent spectral shape and intensity across different spots (Figure 3d), indicating more homogeneous coupling between PBA and ZnO layers in PBA|ZnO than in PBA|ZnO‐RT. These findings highlight the critical role of interfacial interactions (influenced by fabrication conditions)^[^ ^23^ ^]^ in governing ultrafast interfacial CT dynamics.

### 2.3 Functional Characterization of PBA|ZnO Heterostructures

**Figure 4:**
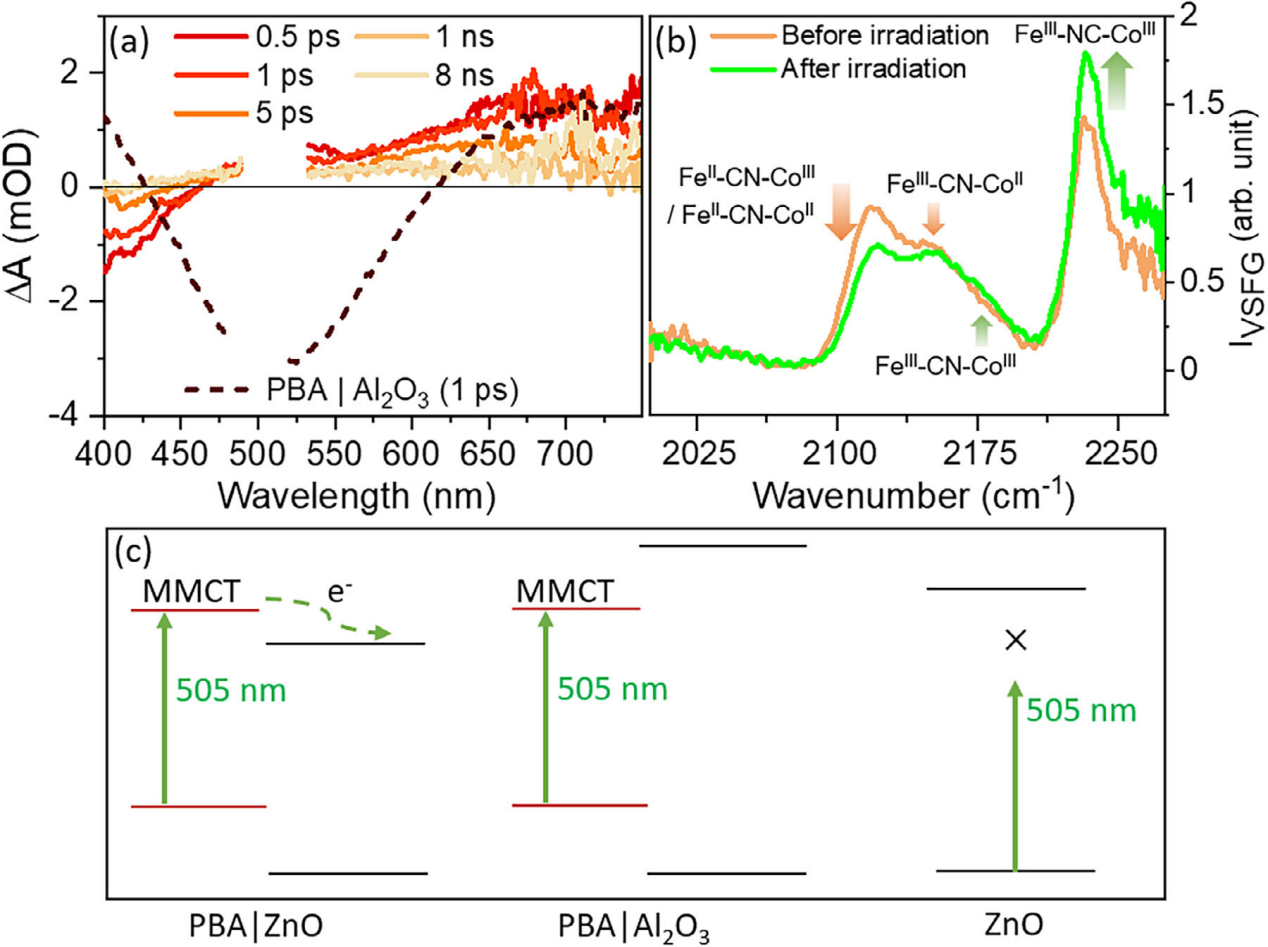
Excitation at 505 nm. a) Ultrafast TA spectra of PBA|ZnO heterostructure with excitation at 505 nm. Excitation energy was 1 µJ/pulse with a spot diameter of 300 µm. The spectrum of PBA|Al_2_O_3_ at 1 ps (dotted line)) is shown for comparison. b) Photo‐irradiated VSFG measurements. VSFG spectrum was recorded before and after irradiation (3 hours) with a 505 nm LED. c) Schematic representation of underlying photophysical processes following an excitation at 505 nm.

PBA|ZnO heterostructures were tested for their photocatalytic water oxidation activities to correlate the rate of CT with the catalytic activity of the heterostructures. The PBA|ZnO and PBA|ZnO‐RT samples in an aqueous solution containing 0.5 M Na_2_S_2_O_8_ (sacrificial agent) were exposed to the output of a solar light simulator, and the oxygen evolution was quantified by gas chromatography (see Figures 3g and S20 and the Supporting Information for experimental details for the photocatalytic experiments and Figures S21–S23 for the postcatalytic analysis of the PBA|ZnO). PBA|ZnO exhibits ∼200% increased activity compared to PBA|ZnO‐RT. Under simulated solar light, the UV and the visible part of the radiation are harvested, respectively, by ZnO and PBA layers (the absorption cross section is higher for ZnO in the UV range and for the CT transition of PBA in the visible range, see Figure 1).^[^ ^34^, ^35^, ^36^, ^37^ ^]^ To probe selectively the effect of visible‐light irradiation, the same experiment was also conducted upon irradiation of the samples with wavelengths >495 nm. These experimental conditions yield an even higher photocatalytic activity, as PBA|ZnO showed a 73% increased oxygen yield, that is, 19.8 µmol of oxygen evolved compared to its activity under solar light irradiation (evolution of 11.2 µmol oxygen). This at first glance counterintuitive result suggests that simultaneous irradiation of the samples with visible and UV light induces additional/faster charge recombination channels, limiting the catalytic efficiency. While the functional characterization presented here shows that the rate of interfacial CT and the catalytic efficiency of the materials are correlated (see Figure 3g, h), the mechanistic details, which drive the superior catalytic efficiency upon visible light irradiation, remain elusive at this point. The following section focuses on the light‐induced CT driving the catalytic process under visible light irradiation (Figure 4a–c).

### 2.4 PBA‐Centered Excitation: Electron Transfer at the PBA|ZnO Interface

We performed TA experiments upon excitation at 505 nm and in situ VSFG spectroscopy under continuous irradiation (505 nm) to study visible light‐driven interfacial CT and subsequent structural changes in the interfacial PBA layer. As mentioned in the previous section, to fully understand the charge dynamics intrinsic to PBA|ZnO, it is crucial to examine the photophysics of ZnO and PBA under identical experimental conditions. Relevant data is provided in the Supporting Information (Figures S12‐S15).

Briefly, upon 505 nm excitation, neither pristine ZnO nor hot water‐treated ZnO (mimicking PBA|ZnO preparation conditions) displays any detectable TA signal (Figures S14‐S15). However, the TA spectrum of PBA|Al_2_O_3_ exhibits a GSB around 500 nm and ESA at wavelengths above 620 nm and below 420 nm (Figure S12). These findings align with Cammarata et al.’s observations of a GSB at 540 nm and ESA at higher and lower wavelengths upon 540 nm excitation of a similar CoFe PBA (in solution).^[^ ^16^ ^]^ They attributed these spectral changes to the Co^III^Fe^II^ to Co^II^Fe^III^ transformation. Their combined transient optical and X‐ray absorption studies suggest a spin transfer (ST) process immediately after excitation in the excited Co^III^ center, leading to a sequential S = 0 → S = 1 → S = 2 spin state transition, which initiates the transient signal buildup within 50 fs.^[^ ^16^ ^]^ This ST further drives intermetallic CT, resulting in the formation of the Co^II^(S = 3/2)Fe^III^(S = 1/2) state within 200 fs.

While comparing the kinetics of these processes, we note some significant differences: unlike the 50 fs buildup observed by Cammarata et al.,^[^ ^16^ ^]^ our measurements show a gradual build‐up of GSB and ESA within 3 to 4 ps (see Figure S12), followed by a 60 ps component and a long‐lived component that persists beyond our experimental window. This timescale is more comparable to Kamioka et al.’s report of CT pair formation within 25 ps after CT excitation.^[^ ^25^ ^]^ As Cammarata et al. studied PBA dispersed in solution, whereas PBA|Al_2_O_3_ (this work) and Kamioka et al.’s work involved immobilized PBA, which provides a more rigid environment and restricts structural rearrangements accompanying CT and ST, we ascribe the different timescales for CT to the differing physical environments of the PBA in the different experiments.

In summary, excitation at 505 nm induces intermetallic CT at PBA|Al_2_O_3_, spectral features of which include a GSB around 500 nm and ESA at wavelengths above 620 nm and below 420 nm. However, measurements with PBA|ZnO under identical excitation conditions show spectral features different from PBA|Al_2_O_3_. In contrast to the GSB of PBA|Al_2_O_3_ at 500 nm, PBA|ZnO exhibits an ESA in the 475–550 nm region (Figure 4a).

Additionally, PBA|ZnO shows an instantaneous GSB at wavelengths below 450 nm, resembling the bleach of ZnO interband transitions (see Figures 2, 4). Kinetic analysis of the ESA at 475 nm revealed that the CT occurs within 1 ps after excitation and does not show any decay within the experimentally accessible delay‐time window of 9 ns (see Figure S16). However, a similar kinetic analysis of PBA|ZnO following 375 nm excitation (upon excitation of ZnO at 375 nm, PBA is oxidized via hole transfer from the VB of excited ZnO, as discussed in the previous section) showed 58% decay of the signal (please also see Figure S16 for comparison). The longer lifetime of the oxidized PBA under 505 nm excitation (as compared to 375 nm excitation) could thus be a contributing factor to the enhanced photocatalytic performance of PBA|ZnO under visible light irradiation.

Herein, we note that the formation of the ESA at 475–550 nm is unique to PBA|ZnO and distinctly differs from the spectral features associated with PBA‐centered ST and CT as discussed above. More importantly, it matches the spectrum of oxidized PBA observed in our UV‐vis spectroelectrochemistry measurements (Figure S4). Hence, we assign this to interfacial electron transfer from excited PBA to the CB of ZnO, resulting in PBA oxidation.

This observation points to the fact that irrespective of the optically excited moiety, that is, ZnO‐centered excitation at λ_pump_ = 375 nm or PBA‐centered excitations at λ_pump_ = 505 nm, a common CT species (oxidized PBA) is formed which absorbs between 475 and 550 nm as reported in literature^[^ ^28^, ^29^ ^]^ and corroborated by our UV‐Vis spectro‐electrochemistry (see Figure S4). However, since the chemical structure of PBA is nonstoichiometric and contains a mixture of neighboring Fe^II^‐CN‐Co^II^, Fe^II^‐CN‐Co^III^, and Fe^III^‐CN‐Co^II^ centers that are differently sensitive to varying redox potentials, the chemical composition of the oxidized PBA (change in composition after CT) has been elusive.^[^ ^13^, ^38^, ^39^ ^]^ To shed light on the compositional changes of PBA, we performed in‐situ VSFG experiments under continuous wave irradiation on the heterostructures at 505 nm. As has been detailed before, in our studies, PBA|ZnO interface consists of Fe^II^‐CN‐Co^II^, Fe^II^‐CN‐Co^III^, Fe^III^‐CN‐Co^II^, Fe^III^‐CN‐Co^III^, and Fe^III^‐NC‐Co^III^ coordination motifs.^[^ ^23^ ^]^ Upon irradiation at 505 nm for 3 hours, an increase in the intensity of the peaks at 2180 cm^−1^ and 2230 cm^−1^ was observed, which can be assigned to the increase in the population of Fe^III^‐CN‐Co^III^ and Fe^III^‐NC‐Co^III^ moieties, respectively, based on the vibrational spectrum of corresponding species as reported previously (Figure 4b).^[^ ^23^ ^]^ At the same time, the intensities of the bands at 2115 cm^−1^ and 2150 cm^−1^ decrease (see Figure 4b). The decreased intensity at 2150 cm^−1^ is attributed to the lowering of the Fe^III^‐CN‐Co^II^ population. The band at 2115 cm^−1^ has, however, contributions from both Fe^II^‐CN‐Co^II^ and Fe^II^‐CN‐Co^III^ motifs.^[^ ^35^, ^40^ ^]^ Thus, the in‐situ VSFG experiments reflect an increase of oxidized PBA motifs majorly through the conversion of Fe^II^‐CN‐Co^II^/Fe^II^‐CN‐Co^III^ to the Fe^III^‐NC‐Co^III^ species at PBA|ZnO interface, which is mechanistically related to light‐induced CT (Figure 4c) across the PBA|ZnO heterostructure following the excitation of either ZnO or PBA.

### 2.5 Electronic Characterization of the Surface

Finally, UV‐Vis diffuse reflectance spectroscopy (UV‐Vis DRS), VB XPS spectroscopy (VB‐XPS), cyclic voltammetry (CV), and Mott‐Schottky (MS) analysis were combined to characterize the electronic states associated with the PBA|ZnO interface. This approach enables us to determine critical parameters, e.g., the bandgap (E_g_), the VB (E_v_), the conduction band (E_c_) of ZnO, and the energy levels of PBA.

**Figure 5:**
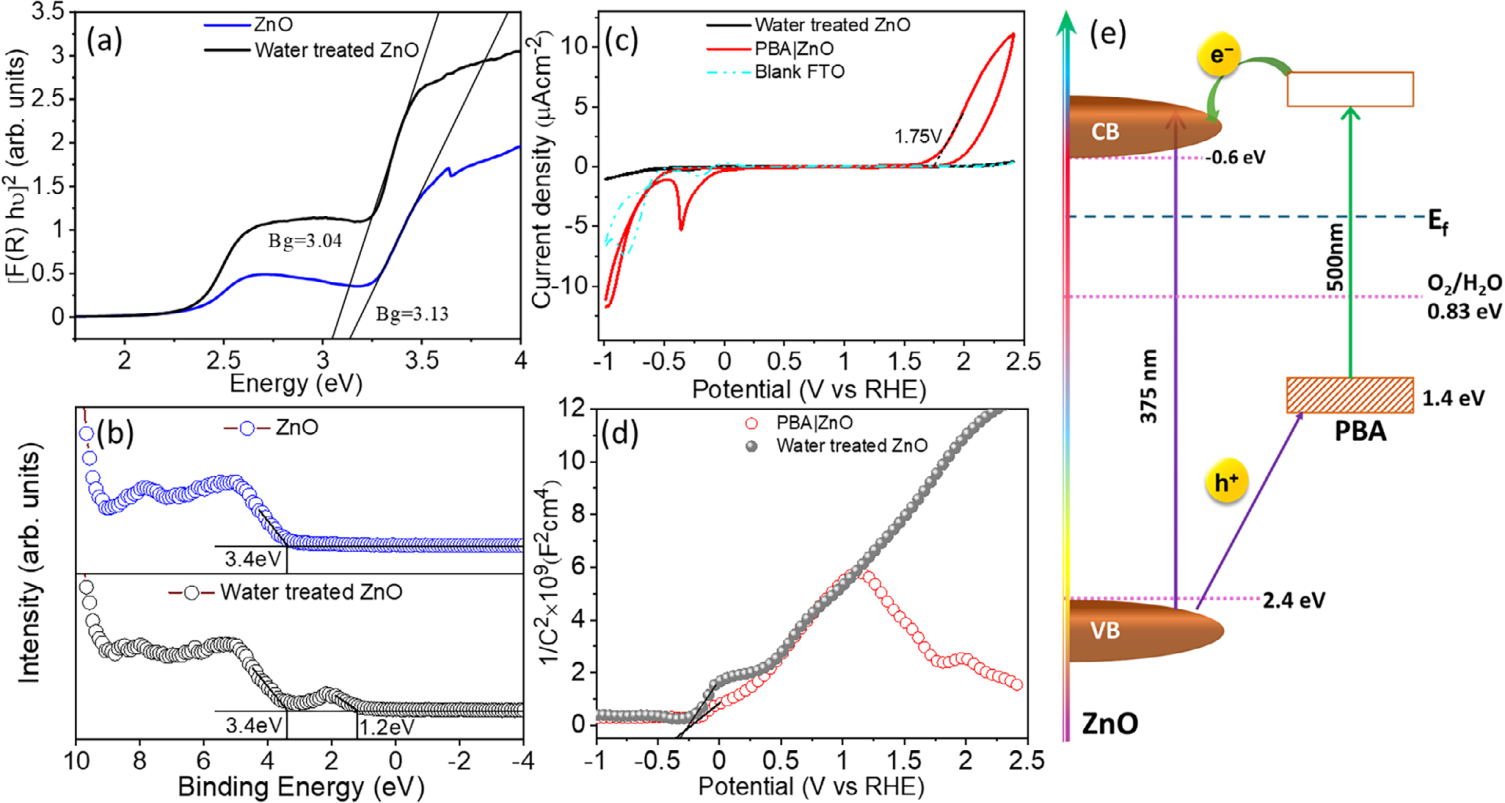
The mechanism of CT during photocatalysis. a) Kubelka‐Munk plots for direct band gap determination derived from diffuse reflectance spectra, and b) VB XPS spectra of ZnO and water‐treated ZnO. c) CV curves and d) Mott − Schottky plots at 500 hz for ZnO and PBA|ZnO in 0.1 M PBS electrolyte (pH 7) at a scan rate of 50 mV/s. The black lines represent the flat band potential values. e) Schematic representation of band energy alignment and CT involved in PB|ZnO‐RT and PB|ZnO for the photocatalytic water oxidation process, the arrow represents the energy axis.

A plot of (αhν)^2^ versus the photon energy (Figure 5a) reveals the electronic bandgap E_g_ of the direct bandgap semiconductor ZnO as 3.13 eV. However, the band gap changes to 3.04 V when ZnO was exposed to the fabrication condition of PBA|ZnO (treating ZnO with water at 75 °C mimicking the fabrication condition of PBA|ZnO, see Supporting Information). The reduced bandgap of ZnO indicates the formation of defects and vacancies upon water treatment.^[^ ^41^, ^42^ ^]^ VB‐XPS measurements corroborate defect formation, as they show a band at 1.23 eV upon water treatment of ZnO, in addition to the band edge at 3.4 eV of the pristine ZnO substrate (Figure 5b). The defect states (DS) induced by hot water treatment (during fabrication of PBA|ZnO) of the ZnO are located between the VB and the CB. The presence of such DS effectively upshifts the VB that makes hole transfer from VB of ZnO to PBA energetically facile.^[^ ^43^ ^]^ These defects primarily arise from the oxygen vacancies in the ZnO lattice and/or surface‐exposed hydroxyl groups resulting from dissociated adsorption of water at higher temperatures.^[^ ^44^, ^45^ ^]^ Such in‐situ modification of the ZnO surface during fabrication of PBA|ZnO allows for strong electronic coupling between PBA and ZnO through the interaction of surface‐exposed ligands of PBA with surface defects (such as exposed “O”) of ZnO.^[^ ^46^, ^47^ ^]^ This electronic interaction also changes the elemental composition of PBA|ZnO as compared to that of PBA|ZnO‐RT as substantiated by the XPS analysis (please see the elemental composition of PBA|ZnO and PBA|ZnO‐RT shown in Table S1). Thus, we cannot rule out the influence of the modified composition of the PBA on the photocatalytic performance of PBA|ZnO. For example, the significantly higher oxygen content, that is, ∼42% in PBA|ZnO as compared to ∼24% in PBA|ZnO‐RT (see Table S1), might reflect the presence of more interstitial water in PBA|ZnO, which is known to add structural stability in Prussian blue during CT.^[^ ^48^ ^]^

Mott‐Schottky analysis determined the flat band potential (V_FB_) for ZnO to be − 0.40 V, which agrees with previous studies (Figure S17).^[^ ^49^ ^]^ Also, after water treatment and formation of PBA|ZnO, V_FB_ remains in the − 0.3 to − 0.4 V range versus RHE (Figure 5d). The positive slope of the Mott‐Schottky plot of ZnO and water‐treated ZnO indicates an n‐type semiconductor, which forms a p‐n junction when coupled with PBA in PBA|ZnO, as evidenced by the V‐shaped Mott‐Schottky plot (Figure 5d).^[^ ^50^ ^]^ The resultant p‐n junction creates a strong electric field in the space charge region and reduces the CT resistance at the interface, thereby facilitating the movement of the charge carrier across the interface.^[^ ^51^ ^]^ We also note that the highly oxidizing hole of ZnO, due to its higher valence band maximum (VBM) of 3.4 eV as determined by VB‐XPS measurements, likely contributes to the superior performance by facilitating hole transfer. The experimental observation that BiVO_4_, with a VBM of approximately 2 eV, requires an applied positive bias of around 0.3 V versus RHE (as reported by Moss et al.)^[^ ^13^ ^]^ to induce hole transfer to PBA, while ZnO can achieve the same process without external bias, strongly supports this notion.

Figure 5e displays the band alignment in the PBA|ZnO heterostructures. The position of the CB (E_CB_) of ZnO is approximated to be 0.6 eV (vs. NHE) as obtained from the flat band potential (V_FB_). The position of the VB is calculated from E_CB_ and Eg (E_g =_ E_VB_ − E_CB_). The estimated E_VB_ is 2.4 eV, which appears to lie between the band edge of bulk ZnO and the surface DS (see above the discussion about the VB‐XPS data). As reported previously by Krzywiecki et al., the band structure of such ZnO thin films is strongly influenced by the depth/thickness‐dependent distribution of carrier/DS.^[^ ^52^ ^]^ Thus, different techniques having different probe depths produce slightly altered band positions. The HOMO levels of PBA, that is, 1.75 eV (vs. NHE), were extracted from cyclovoltammetry (Figures 5c and S18).

## 3 Conclusion

This study highlights the interplay between structure and CT efficiency at the interface of Co‐Fe Prussian blue analogue|ZnO (PBA|ZnO) heterostructures, which plays a key role in their photocatalytic activity. TA spectroscopy and time‐resolved VSFG spectroscopy revealed that light‐induced CT occurs as fast as one picosecond when the interfacial composition is engineered via a facile synthetic protocol. This is of particular importance since intermolecular interactions in the interface make even kinetically challenging structural rearrangements feasible in a rigid interfacial environment, which is key to the ultrafast interfacial CT. We also show that the interfacial CT, boosted by the structural co‐operativity in the interface, enhances the photocatalytic activity significantly.

Furthermore, the comparatively superior performance of PBA|ZnO can be attributed to a combination of factors: highly efficient interfacial CT, which promotes the activation of Co sites for the catalysis, and the higher amount of interstitial water that contributes to the structural stability of PBA during CT.

In conclusion, the findings present a cornerstone for the fundamental understanding of the key factors that govern the interfacial charge dynamics. The concept of “interfacial co‐operativity” can be expanded to rational interfacial engineering of heterostructures for energy conversion and storage systems, where interfacial charge separation controls the performance of the device.

## Supporting Information

The Synthesis details of the heterostructures, description of experimental set up and additional results experimental results concerning TA, TR‐VSFG, UV‐Vis SEC, XPS, Photocatalytic and Electrochemical measurements are included in the Supporting Information. The authors have cited additional references within the Supporting Information.^[^ ^53^, ^54^, ^55^, ^56^ ^]^

## Conflict of Interest

The authors declare no conflict of interest.

## Supporting information



Supporting Information

## Data Availability

The data that support the findings of this study are available in the supplementary material of this article.
