# Supplementary material for: Interfacial Co‐Operativity Enables Ultrafast Charge Transfer Within the Co‐Fe Prussian Blue Analogue|Zno Heterostructure
Source: Chemistry. 2025 Aug 22;31(53):e01696. doi: 10.1002/chem.202501696 (PMC12451413; doi:10.1002/chem.202501696)
Supplement: Supplementary file 1 — Supporting Information [file CHEM-31-e01696-s001.pdf]

Interfacial co-operativity enables ultrafast charge transfer within the Co-Fe Prussian blue analogue |ZnO heterostructure

Ratnadip De[a] [b], Ruby Phul[c], Marius Hermesdorf [d], [e], Jyoti Bisht[a] [b], Annett Gawlik[a], Martin Oschatz[d], [e],[f] , Ferdi Karadaş*[a] [b] [c] [g], and Benjamin Dietzek-Ivanšić*[a] [b] [d]

[a] R. De, J. Bisht, A. Gawlik, Prof. F. Karadaş and Prof. B. Dietzek-Ivanšić
Department of Functional Interfaces, Leibniz Institute of Photonic Technology Jena,
Albert-Einstein-Strasse 9, 07745, Jena, Germany

[b] R. De, J. Bisht, Prof. F. Karadaş and Prof. B. Dietzek-Ivanšić
Institute of Physical Chemistry, Friedrich Schiller University Jena,
Helmholtzweg 4, 07743, Jena, Germany

[c] Dr. R. Phul, Prof. F. Karadaş
Department of Chemistry, Main Campus, Bilkent University,
06800, Ankara, Turkey.

[d] M. Hermesdorf, Prof. Dr. Martin Oschatz, Prof. B. Dietzek-Ivanšić
Center for Energy and Environmental Chemistry, Friedrich Schiller University Jena,
Philosophenweg 7a, 07743, Jena, Germany

[e] M. Hermesdorf, Prof. Dr. Martin Oschatz,
Institute for Technical Chemistry and Environmental Chemistry, Friedrich Schiller University Jena,
Philosophenweg 7a, 07743, Jena, Germany

[f] Prof. Dr. Martin Oschatz,
Helmholtz Institute for Polymers in Energy Applications Jena (HIPOLE Jena), Lessingstraße 12-14, 07743 Jena, Germany

[g] Prof. F. Karadaş
UNAM – National Nanotechnology Research Center, Bilkent University,
Ankara 06800, Turkey.

E-mail: benjamin.dietzek@leibniz-ipht.de (B.D-I)
E-mail: karadas@fen.bilkent.edu.tr (F. K.)

Experimental details.

Fabrication of PBA|ZnO

ZnO substrates were prepared by atomic layer deposition (ALD), which produces atomically smooth ZnO surfaces with controlled thicknesses. The thickness of the resulting ZnO film was 50 nm. For the preparation of PBA|ZnO, first, an aqueous solution of PBA was prepared by dropwise adding 2.5 ml of 0.0375 M of $Co(NO_3)_2{\cdot}6H_2O$ to 25 ml of 0.025 M of $K_3Fe(CN)_6$ with continuous stirring. Following this, the ZnO substrates were vertically immersed into the solution, and the setup was kept at 75 °C for 12-14 hours. Afterward, the PBA|ZnO sample was removed, rinsed with deionized water, and dried under N_2.

For the preparation of PBA|ZnO-RT, the same procedure was followed, however, instead of 75 °C, the fabrication was done at room temperature.

For controlled experiments, water-treated ZnO substrates were prepared by vertically immersing ZnO substrates in water at 75 °C for 12-14 hours.

Materials Characterization

The phase analysis of the pristine and post-catalysis samples was determined by X-ray diffraction studies using a Pan analytical X'PertPro multipurpose X-ray diffractometer (MPD) with Cu Kα X-ray radiation (λ = 1.5418 Å). The diffraction patterns were recorded in the 2-theta range of 10-80° with a step size of 0.01. The diffraction peaks shown in Figure 1b could be indexed to the hexagonal wurtzite structure of zinc oxide. The low-intensity peaks at 16.2°, 19.5°, and 21.7° confirm the formation of a thin layer of CoFe-PB on the ZnO substrates (Figure 1b)[1]. Infrared (IR) spectra were recorded with a Bruker Alpha Platinum-ATR spectrometer with 2 cm^{-1} resolution in a wavenumber range 4000-400 cm^{-1}. The morphological features of the pristine and post-catalytic samples were examined by a scanning electron microscope (SEM, FEI-Quanta 200 FEG ESEM).

The optical characterization was done using UV-vis diffuse reflectance spectroscopy (UV-vis DRS) via Cary 5000 UV-vis–NIR spectrometer equipped with a Varian Cary 2500 internal diffuse reflectance (DR) accessory.

Transient absorption spectroscopy

Ultrafast transient absorption (TA) measurements were performed using a custom-built experimental setup. A Ti:Sapphire regenerative amplifier (Coherent, Astrella) was used that generates ~85 fs pulses centered at ~800 nm with a 1 kHz repetition rate. The laser output was split, with one portion focused into a rotating CaF_2 crystal to produce a white-light continuum spanning 300-800 nm. This continuum was divided into a reference and probe beam. The second laser portion was directed into an optical parametric amplifier (TOPAS prime, Light Conversion) to create tunable pump pulses. For this study, 375 nm and 505 nm pump pulses were employed. A mechanical chopper reduced the pump pulse repetition rate to 500 Hz to enable pump-on and pump-off measurements. The relative polarization of pump and probe beams was set to the magic angle (54.7°) using a Berek compensator and polarizer.

Probe and reference spectra were recorded with a Czerny-Turner spectrograph (Princeton Instruments, SP2150) equipped with CCD detectors (Pascher Instruments AB).

For the transient absorption measurements freshly prepared and dried (under N_2) heterostructures (PBA|ZnO , PBA|ZnO-RT and PBA|Al_2O_3) were used.

VSFG spectroscopy

A detailed description of the VSFG experimental setup can be found in our previous work.[2] In brief, we employed a Ti:Sapphire regenerative amplifier (Astrella, Coherent Inc.) generating 85 femtosecond pulses centered at ~800 nm with a pulse power of 5 mW and a repetition rate of 1 kHz, output power 5 W. 2 W of this fundamental beam was spectrally narrowed to approximately 0.62 nm (fwhm) using a 4f pulse shaper to serve as the visible beam for VSFG interaction. 2 W from the remaining fundamental beam was converted into a mid-infrared beam through optical parametric amplification (TOPAS Prime, Light Conversion) and subsequent difference frequency generation (NDFG, Light Conversion). The visible and mid-infrared pulses were spatially and temporally overlapped at the sample, positioned slightly off the beam focus to prevent damage. The resulting sum frequency generation (SFG) signal was spectrally dispersed and detected by a cooled CCD camera (Back-thinned FFT-CCD-Hamamatsu). All measurements were conducted in a PPP polarization configuration, where P represents the polarization of the sum frequency, visible, and infrared beams. The remaining 1 W of the fundamental beam was directed towards an optical parametric amplifier (TOPAS prime, Light conversion, Lithuania) to generate visible pump pulses for TR-VSFG measurements.

Time-resolved vibrational sum frequency generation (TR-VSFG) spectroscopy combines visible and infrared laser pulses with an additional pump pulse. In this work, the 375 nm pump pulse triggers an electronic transition within the system. Subsequently, the VSFG interaction, induced by the simultaneous arrival of the visible and infrared pulses, probes the vibrational modes of the perturbed interface.

TR-VSFG signal was recorded under the pump on and pump off condition and then the difference spectrum of pump on and pump off was calculated. For this, an optical chopper was synchronized with a galvo-mirror and both of them were triggered by the source laser. The optical chopper blocked every second pump pulse to produce pump on and pump off

VSFG signal which was then directed to the upper and bottom panels of the CCD detector by the oscillating galvo-mirror (Figure S1).

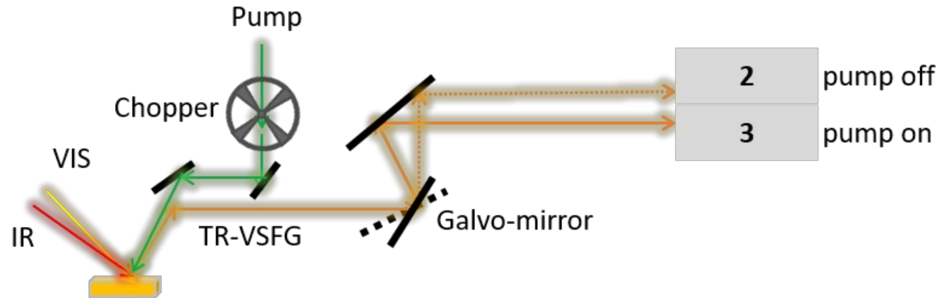

Figure S1. Schematic representation of experimental setup for TR-VSFG measurements.

For the photo irradiated VSFG measurements, a collimated LED (For the collimation, an adjustable collimation Adapter with Ø2" Lens was used) with center wavelength at 505 nm was used as Illumination Source. VSFG signal was recorded each 20 minutes under the illumination. During these measurements 1 cm^2 of the sample area was exposed to the illumination and the output power of the light source was set at 10 mW.

Electrochemical Measurements

The electrochemical measurements were done by a Gamry Instruments Interface 1000 potentiostat/galvanostat. A homemade three-electrode cylindrical cell was used, with Ag/AgCl (3.5 M KCl) as the reference electrode, a Pt wire as the counter electrode, and ZnO or PBA|ZnO coated FTO substrates as the working electrode. Before electrode preparation, the FTO substrates were cleaned by ultrasonication for 15 min each in basic soap solution, deionized water, acetone, and isopropanol and then air dried at 100 °C for 1 h. All the experiments were performed in 0.1 M phosphate buffer solution (PBS; pH 7) containing 0.1 M KNO_3 at room temperature. Before every experiment, the electrolyte was bubbled with N_2 gas for 15 min to remove dissolved O_2. Cyclic voltammetry (CV) measurements were conducted under dark conditions at a scan rate of 50 mV s^{-1} (unless mentioned otherwise). The Mott−Schottky analysis was carried out at 500 Hz frequency in dark conditions to estimate the flat band position of the samples using the following equation:

$$\frac{1}{C^2} = \frac{2}{\varepsilon_0 \varepsilon_r e N_D}\left[V - V_{FB} - \frac{k_B T}{e}\right]$$

where C represents the interfacial capacitance, ε_0 is the vacuum permittivity, ε_r is the dielectric constant of SC, N_D shows the carrier density, V is the applied voltage, k_B is the

Boltzmann's constant, *T* represents the absolute temperature, and *e* stands for the electronic charge. The flat band potential (V_{FB}) was calculated by plotting the $1/C^2$ versus applied potential (V), represented by the potential at which the linear section crosses the horizontal axis. The CB edge position could also be defined by MS plots through the following equation:

$$\Delta E = E_{CB} - E_{FB}$$

where E_{CB} and E_{FB} are conduction and flat band potentials, respectively.

UV-vis spectroelectrochemistry

UV-Vis spectroelectrochemistry measurements were performed at room temperature under ambient condition in a custom-built three-electrode quartz cell with an optical path length of 1 cm. The UV–vis spectra were recorded with a SPECORD S600 spectrophotometer, while electrochemical measurements were run using a BioLogic SP50 potentiostat. For the electrochemical experiments, PBA|ZnO composite was prepared on an ITO substrate, which was used as the working electrode. Pt wire and Ag/AgCl were used as counter electrodes and reference electrodes respectively. 0.1 M $TBABF_4$ dissolved in DCM was used as electrolyte. Cyclic voltammogram (CV) of PBA|ZnO electrode shows an increase in current at potentials above 0.6 V vs. Ag/AgCl, which is indicative of PBA oxidation (please see Figure S3). Following the CV, UV-Vis spectrum was recorded before, during, and after controlled potential electrolysis at 1. 2 V. UV-vis spectrum that was recorded without any applied potential was used as reference spectrum for calculating the differential absorption spectrum.

XPS measurements

The samples were analyzed using a K-alpha spectrometer (Thermo Scientific) in a high vacuum environment. They were attached to the sample holder with copper tape. To counteract charging effects, a flood gun was employed, calibrated to the carbon-carbon double bond peak at 284.8 electron volts with a tolerance of 0.1 electron volts. Consequently, the spectra were not adjusted to a carbon 1s peak at 284.8 electron volts, as potential shifts in binding energies due to chemical alterations could not be ruled out. The analysed area was 400 micrometres in diameter. For overall composition, survey spectra were acquired with a one electron volt step size and a pass energy of 100 electron volts. Detailed spectra of carbon, nitrogen, oxygen, zinc, iron, and cobalt were obtained with a step size of 0.05 electron volts, accumulating five scans for the first four elements and ten scans for the latter two. All high-

resolution spectra were processed using the Avantage v6.6.0 software from Thermo Fisher Scientific.

The energy of the valence band maxima (VBM) near the Fermi level of the samples was measured by the Valence Band XPS spectra (VB-XPS) (i.e., E_F-E_{VBM}, considering E_F~0)[3] by using a Thermo Scientific K-Alpha X-ray photoelectron spectrometer system equipped with an Al Kα micro focused monochromator source (1486.6 eV) operating at 400 µm spot size and accompanied by a flood gun for charge neutralization.

Photocatalytic O_2 Evolution

The photocatalytic experiments were carried out in a photoelectrochemical cell (volume = 14 mL) designed by Specson Instruments (Specsoninstruments.com) at room temperature. In a typical experiment, the catalyst-coated 2.5x2.5 cm^2 glass substrate was inserted in the reactor, and the reactor was filled with 9.5 ml of 0.5 M aqueous solution of $Na_2S_2O_8$ (pH 7). The reaction solution was purged by bubbling N_2 gas and kept in the dark for 30 min to maintain the equilibrium with magnetic stirring. A 300 W Xe lamp (AM 1.5 global filter, 65000 lux) was used as the light source. 1.88 cm^2 are of the catalyst coated substrate was exposed to the light. For the photocatalytic experiments under visible light, an additional 495 nm cutoff filter was used in the same set up (resulting illuminance 36400 lux). The amount of O_2 generated was determined at every 1 h intervals by injecting 100 µL of the reactor headspace gas into a gas chromatograph (Agilent 7820A GC, with molecular sieves column and a TCD detector). Ar was used as the carrier gas. Two rubber septa were used to seal the reactor, where the space between them was purged with N_2 gas to avoid a possible O_2 leak during the measurements.

Supporting Figures.

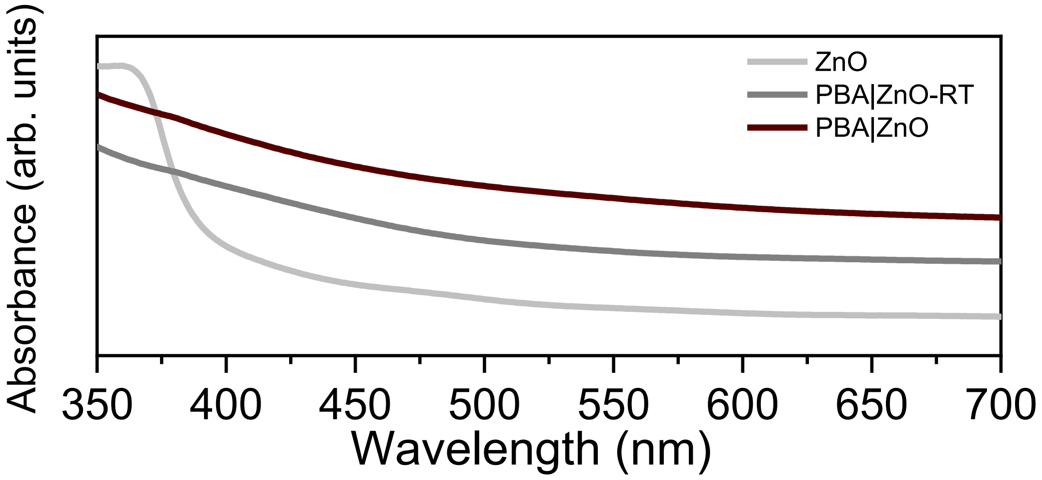

Figure S2. Steady-state absorption spectrum of ZnO, PBA|ZnO-RT, and PBA|ZnO. ZnO exhibits absorption maxima at 362 nm. However, broad/ unstructured spectral shape and excessive light scattering by the adsorbed PBA impede a quantitative analysis of the spectrum of PBA|ZnO and PBA|ZnO-RT.

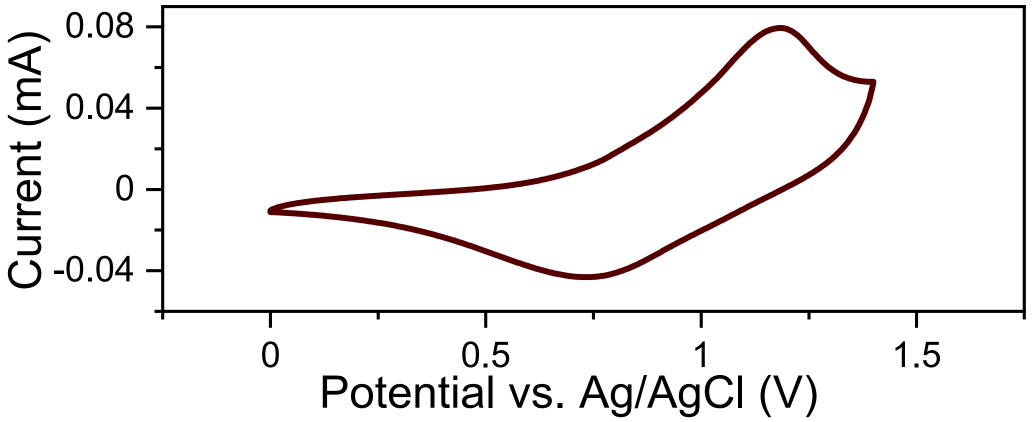

Figure S3. A cyclic voltammogram of PBA|ZnO, prepared on an ITO substrate. Scan rate 10 mV/sec. The CV clearly shows an increase in current at potentials above 0.6 V vs. Ag/AgCl, which is indicative of PBA oxidation.

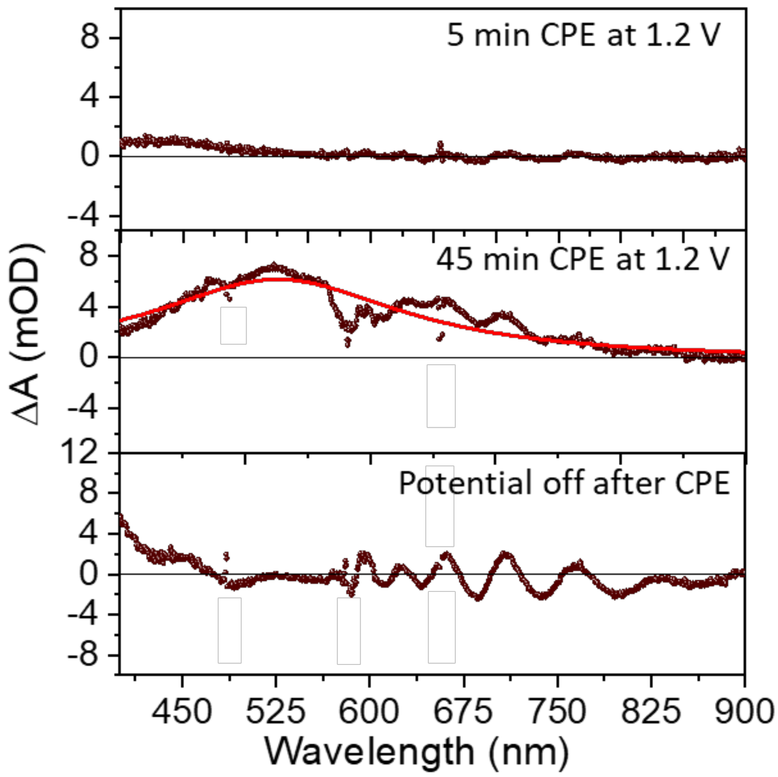

Figure S4. Differential absorption spectra of PBA|ZnO, recorded during controlled potential electrolysis (CPE) at 1.2 V vs. Ag/AgCl after 5 and 45 minutes, revealed the formation of oxidized PBA. Upon removal of the applied potential, this band disappeared. The differential absorption spectra were obtained by subtracting the absorption spectrum of the sample without any applied bias (recorded under otherwise identical experimental conditions) from the spectra recorded during CPE.

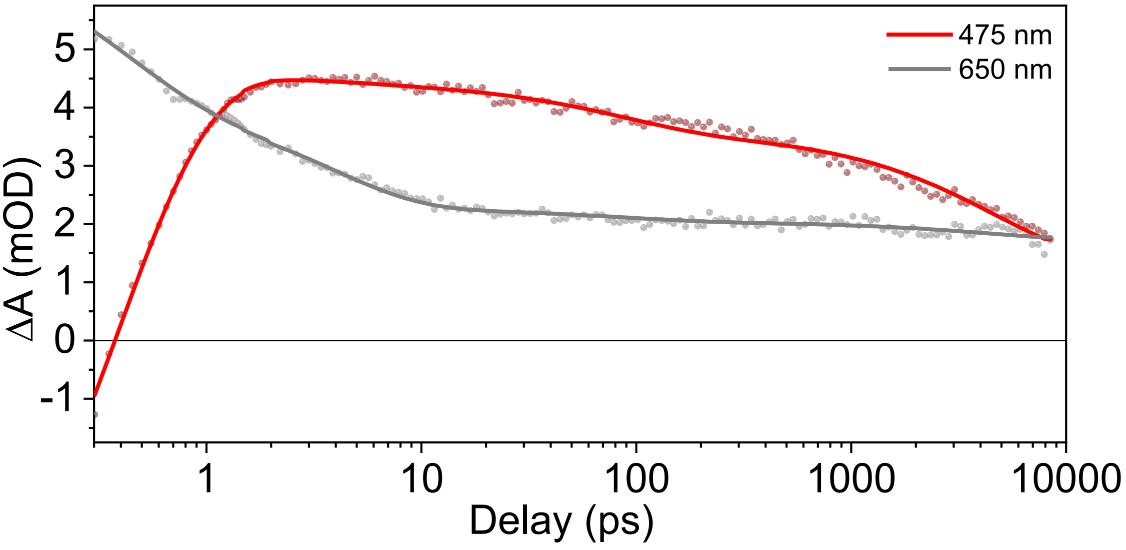

Figure S5. Transient absorption kinetics of PBA|ZnO at 475 nm and 650 nm show that the ultrafast formation of excited state absorption at 475 nm (oxidation of PBA as a result of hole transfer from ZnO to PBA) is accompanied by a simultaneous decrease in absorption at 650 nm. This process is unique to the sample PBA|ZnO.

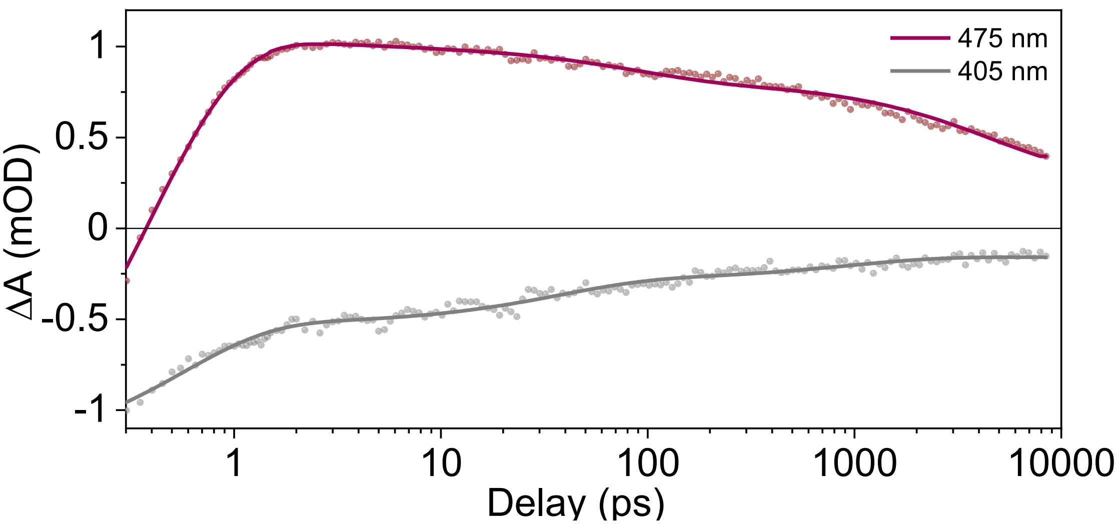

Figure S6. Normalized (at the absolute maximum) transient absorption kinetics of PBA|ZnO at 475 nm and 405 nm show that the ultrafast formation of excited state absorption at 475 nm (oxidation of PBA as a result of hole transfer from ZnO to PBA) matches the recovery of ground state bleach of ZnO at 405 nm.

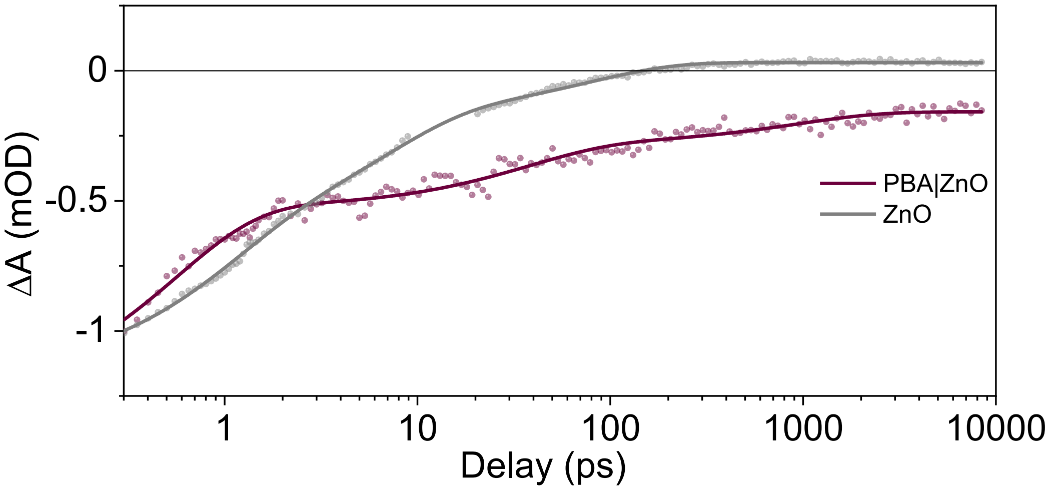

Figure S7. Transient absorption kinetic analysis at 405 nm of PBA|ZnO and ZnO, following 375 nm excitation, highlights the difference in ground-state recovery between bare ZnO and PBA|ZnO. At initial time delays, PBA|ZnO exhibits an ultrafast component (0.3-0.4 ps), which aligns with the 475 nm signal buildup shown in Figure S4. This ultrafast component (which is absent in bare ZnO) can be attributed to interfacial charge transfer between PBA and ZnO. Furthermore, at longer time delays, PBA|ZnO displays a significantly slower (as compared to bare ZnO) recovery of the ground-state bleach compared to bare ZnO. This can be associated with slower recombination of charge carriers at the PBA|ZnO since the charge carriers are separated across the interface.

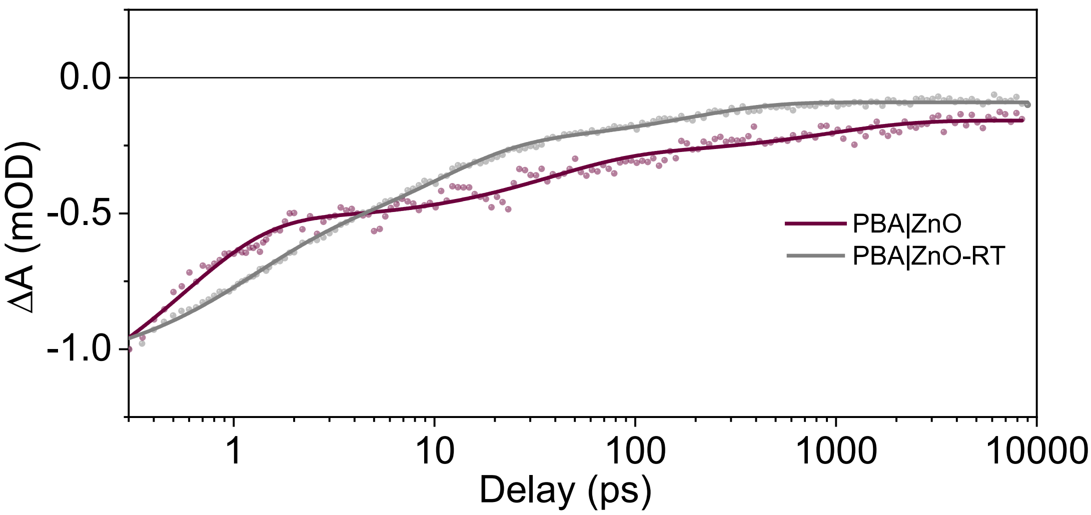

Figure S8. Transient absorption kinetic analysis at 405 nm of PBA|ZnO and PBA|ZnO-RT following excitation at 375 nm. At initial time delay, faster decay was noted for PBA|ZnO which indicates ultrafast transfer of hole from VB of ZnO to the PBA, which was slower in the case of PBA|ZnO-RT.

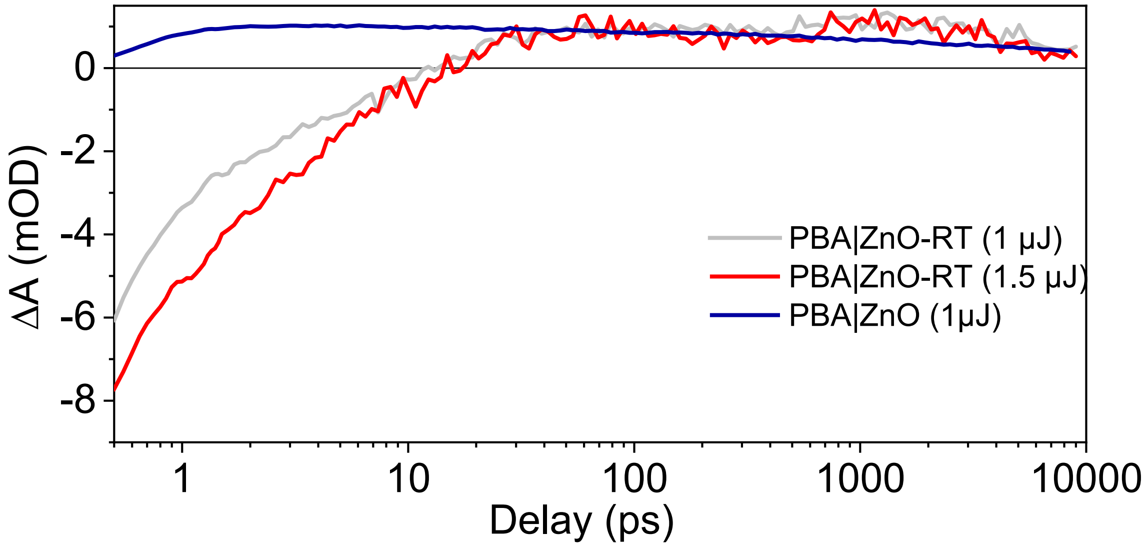

Figure S9. TA kinetics (normalized at maximum differential absorption) at 475 nm showing the formation of oxidized PBA in PBA|ZnO-RT and PBA|ZnO. Even at higher pump fluence the CT is much slower for PBA|ZnO-RT as compared to that of PBA|ZnO.

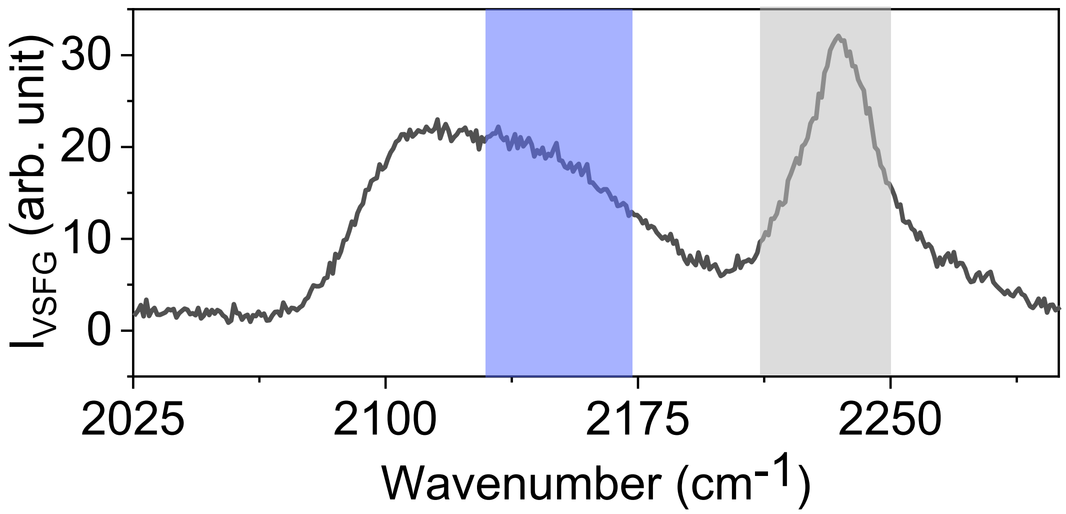

Figure S10. Steady state VSFG spectrum of PBA|ZnO. Integrated kinetics of the highlighted regions (Co^{II}-NC-Fe^{III} -blue and Co^{III}-CN-Fe^{III} - grey) were taken for investigating the CT induced modulation of VSFG signal.

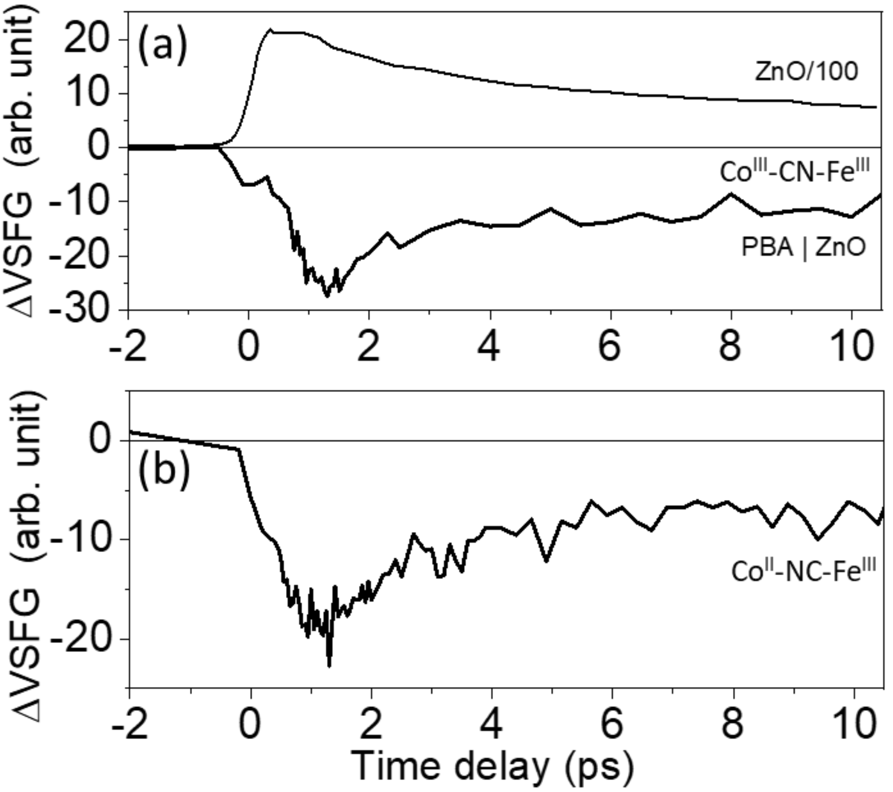

Figure S11. (a) TR-VSFG signal of ZnO and PBA|ZnO showing different transient signal. TR-VSFG signal of ZnO can be attributed to the surface trapped hole[4] following the excitation at 375 nm, however, in PBA|ZnO owing to the transfer of the hole to PBA layer an interfacial electric field is generated that cause formation of negative TR-VSFG signal within ~1 ps. It is important to note here that such modulation of the VSFG signal is not specific to a vibrational band, as it originates from the electric field generated from separated electrons and holes, and thus affects the whole spectral region equally. For PBA|ZnO, we choose to integrate the vibrational band associated with Co^{III}-CN-Fe^{III} since owing to its distinct position (which does not overlap with any other bands), it offers the best signal-to-noise ratio. (b) TR-VSFG signals of PBA|ZnO, integrated over the vibrational band Co^{II}-NC-Fe^{III}. Comparably similar kinetic feature associated with the two different vibrational bands (Co^{II}-NC-Fe^{III} and Co^{III}-CN-Fe^{III}) substantiate the electric field induced modulation of the VSFG signal.

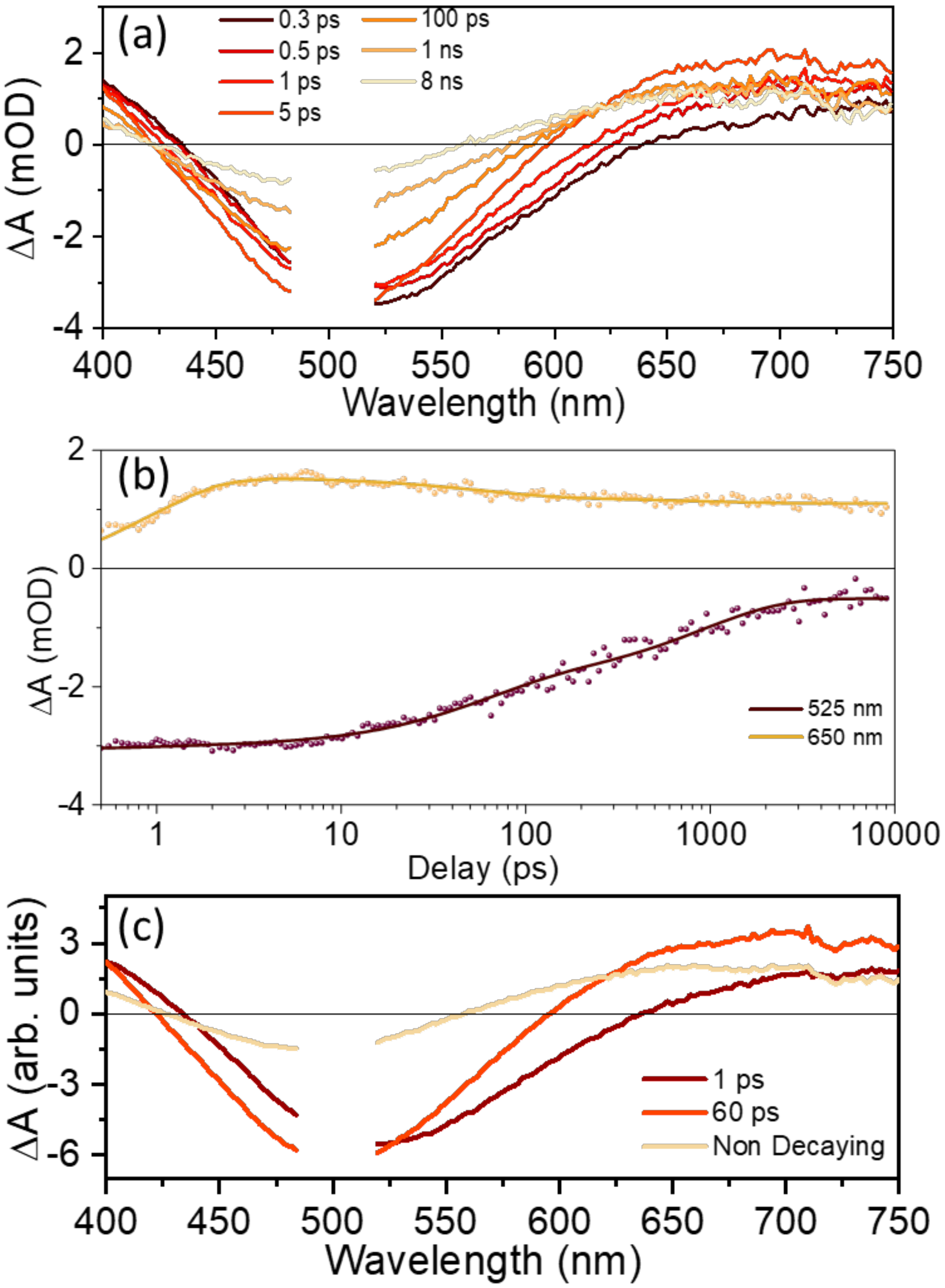

Figure S12. (a)TA spectra, (b) kinetics at selected wavelengths (c) spectra of species associated with the first two decay components and long-lived component following the excitation of PBA|Al_2O_3 at 505 nm.

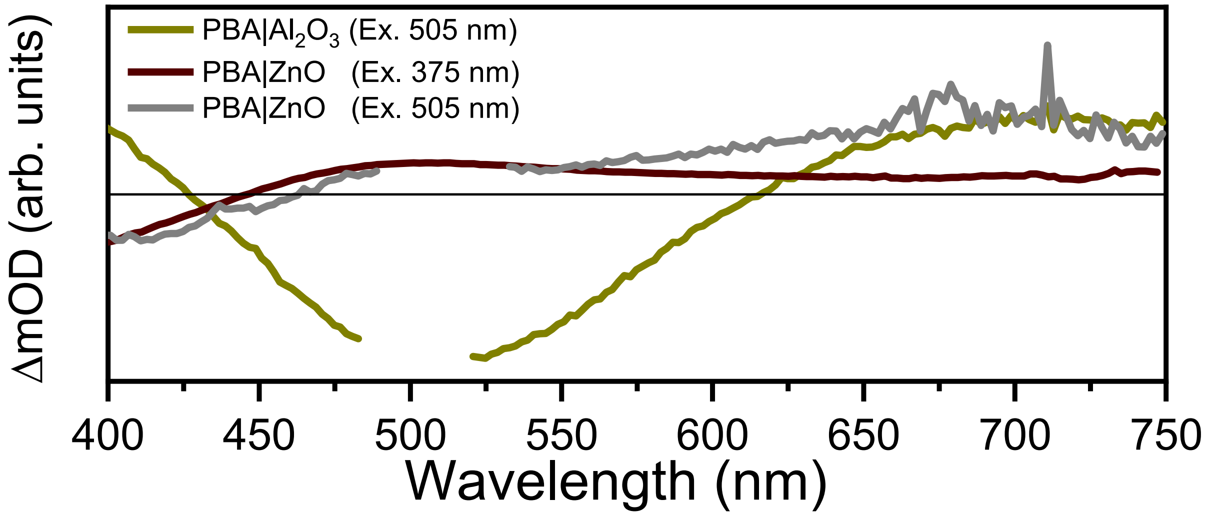

Figure S13. Transient absorption spectra of PBA|ZnO at 1 ps following excitation at 375 nm (brown line) and 505 nm (gray line) are shown (normalized at 500 nm). For 505 nm excitation, the ESA band of PBA|ZnO at probe wavelengths > 620 nm resembles the spectral features observed for the reference sample PBA|Al_2O_3 (green line, scaled to the spectral feature of PBA|ZnO at 700 nm, under the same excitation condition). However, PBA|ZnO exhibits an additional ESA band in the 475-550 nm region, which is absent in the spectra of reference sample (PBA|Al_2O_3) but matches the spectral feature of oxidized PBA (also formed after 375 nm excitation of PBA|ZnO). The graph demonstrates that, regardless of the optically excited moiety, i.e., ZnO-centered excitation at 375 nm or PBA-centered excitation at 505 nm, a common CT species is formed, absorbing between 475 and 550 nm.

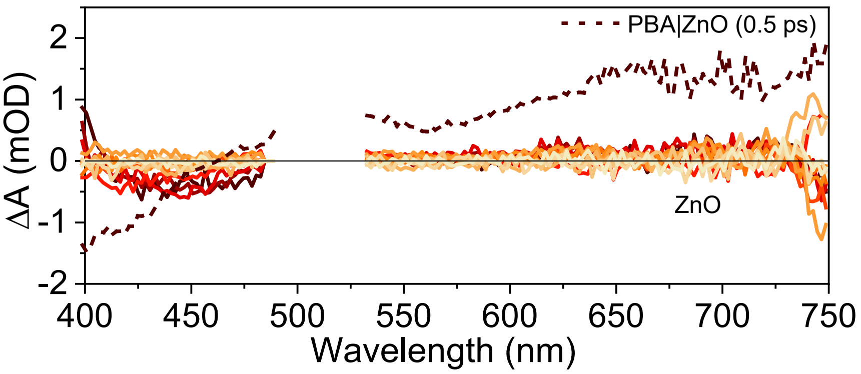

Figure S14. TA spectra of ZnO following excitation at 505 nm. The signal is within the resolution limit. TA spectrum of PBA|ZnO is shown for comparison.

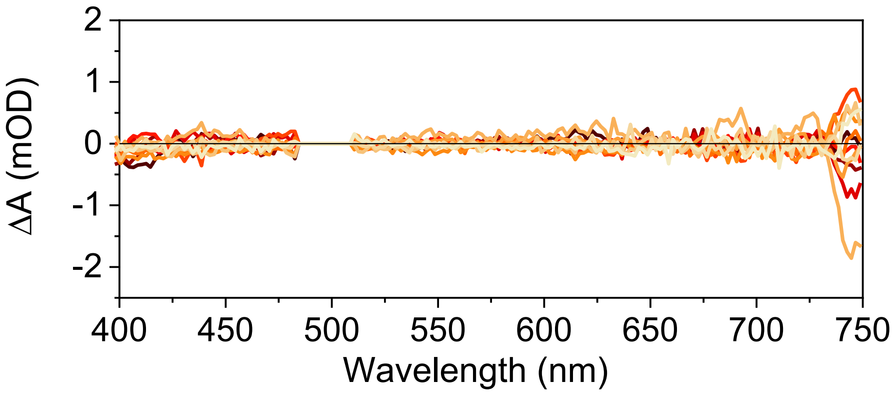

Figure S15. TA spectra of ZnO after water treatment at 75°C following excitation at 505 nm. The signal is within the resolution limit.

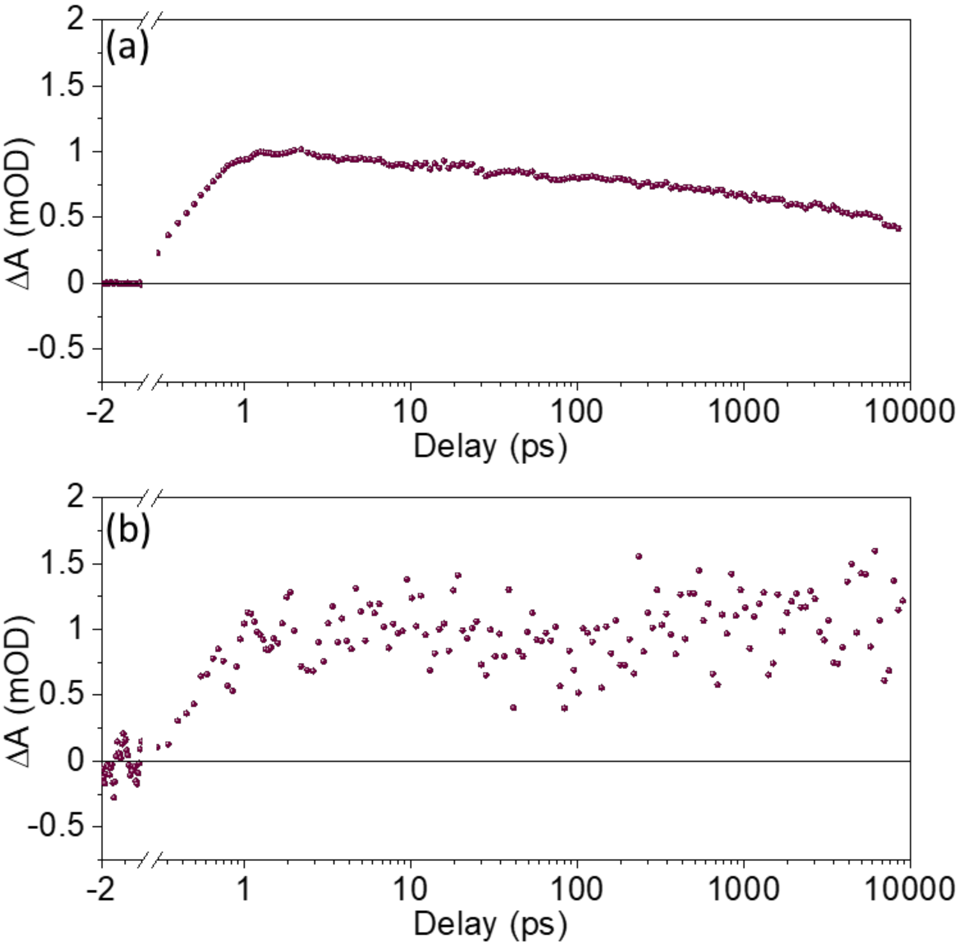

Figure S16. Kinetic trace at 475 nm after exciting PBA|ZnO with (a) 375 nm pump (ZnO centered excitation) and (b) 505 nm pump (PBA centered excitation). Kinetic analysis following the excitation at 505 nm revealed that the CT occurs within 1 ps after excitation and does not show any decay within the experimentally accessible delay-time window of 9 ns. However, a similar kinetic analysis of PBA|ZnO following 375 nm excitation (upon excitation of ZnO at 375 nm, PBA is oxidized via hole transfer from the VB of excited ZnO) showed 58% decay of the signal. The longer lifetime of the oxidized PBA under 505 nm excitation (as compared to 375 nm excitation) could thus be a contributing factor to the enhanced photocatalytic performance of PBA|ZnO under visible light irradiation.

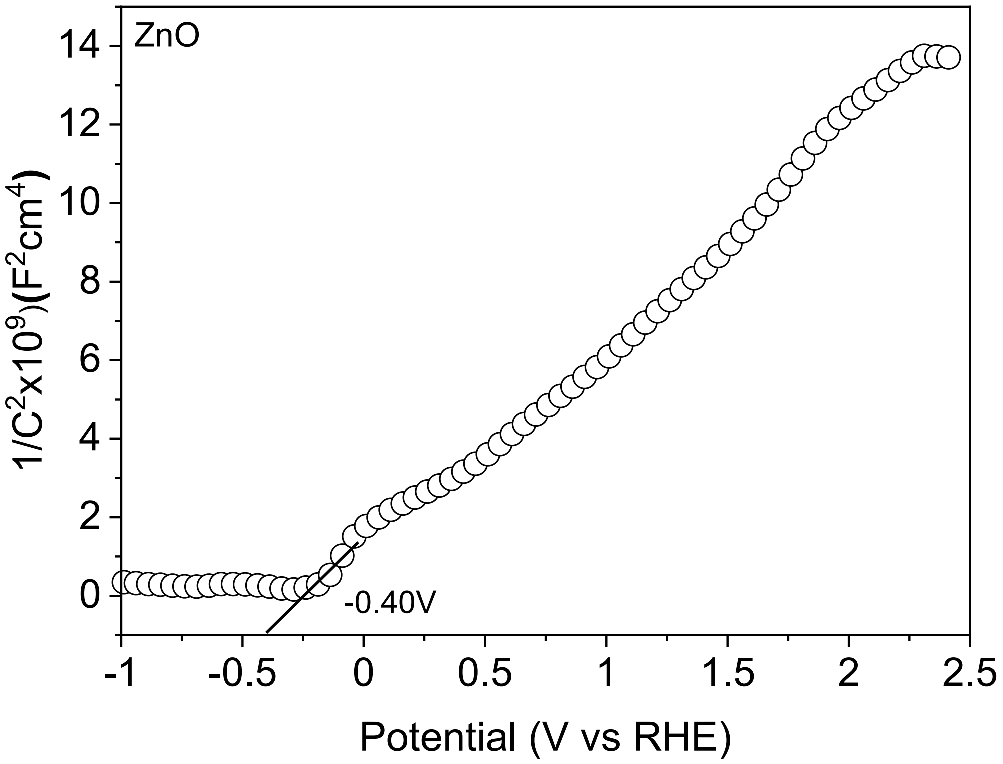

Figure S17. Mott–Schottky plots at 500 hz for ZnO in 0.1 M PBS electrolyte (pH 7) at the scan rate of 50 mV/s. The red lines represent the flat band potential values.

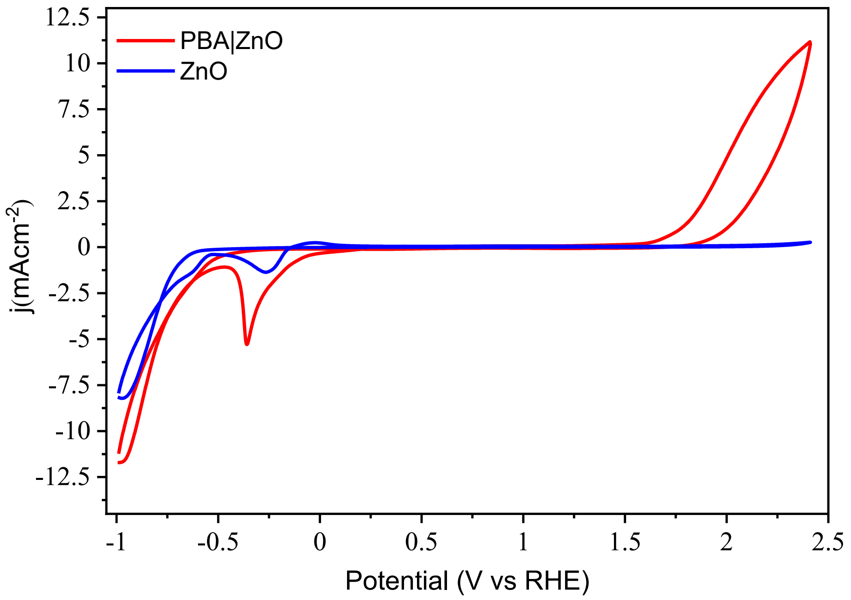

Figure S18. Cyclic voltammogram of ZnO and PBA|ZnO in 0.1 M PBS (pH=7.05) at the scan rate of 50 mV/s.

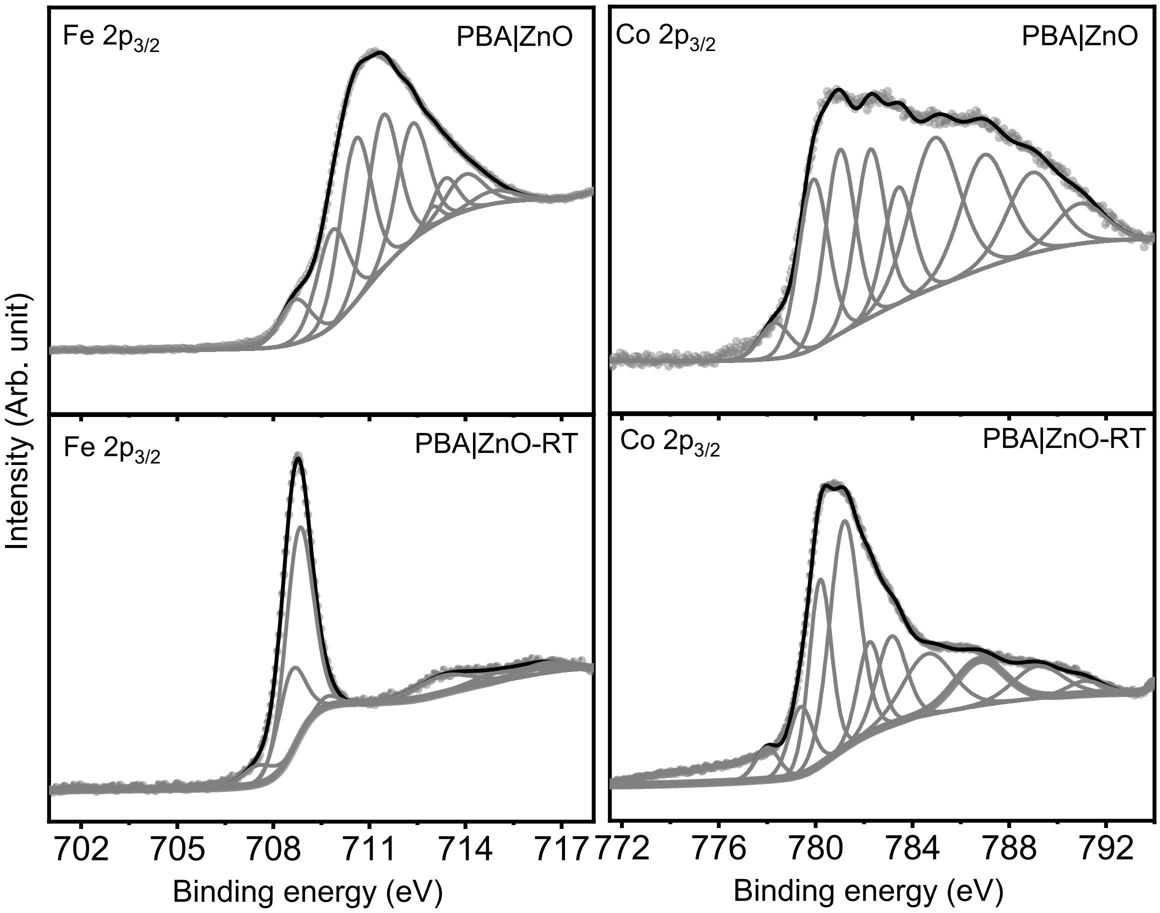

Figure S19. HR-XPS spectra of Fe2p$_{3/2}$ and Co2p$_{3/2}$ of PBA|ZnO and PBA|ZnO-RT. Significantly broadened XPS signals of Fe and Co of PBA|ZnO indicate a modified electronic environment of PBA molecules owing to a stronger interaction with the ZnO surface, as compared to that of PBA|ZnO-RT. Spectral change indicates differently oxidized Co (e.g. Co^{II} and Co^{III}) and Fe, which is also evident from the elemental analysis (lowering the amount of K^+ indicate oxidation of Co and Fe centers in PBA).

Table S1. Elemental analysis of PBA|ZnO and PBA|ZnO-RT using XPS

	PBA|ZnO (in %)	PBA|ZnO-RT (in %)
C1s	46.3	47.4
K2p	0.2	2
O1s	41.8	24.0
N1s	1.8	14.6
Zn2p	0.2	2.5
Fe2p	6.2	1.5
Co2p	3.4	7.7

Elemental analysis revealed a decrease in the amount of K^+ ion in PBA|ZnO, indicating a higher presence of Co and Fe in +3 oxidation state. HR XPS spectrum also indicates the same, as evidenced by the broadening of the spectrum towards higher energy region. Furthermore, an increased amount of oxygen indicates the presence of interstitial water in PBA|ZnO, which was prepared at high temperatures (75°C). Different elemental compositions, especially the presence of oxidized Fe and Co in PBA|ZnO substantiate the stronger substrate-molecule interaction in PBA|ZnO, as also discussed in the manuscript.

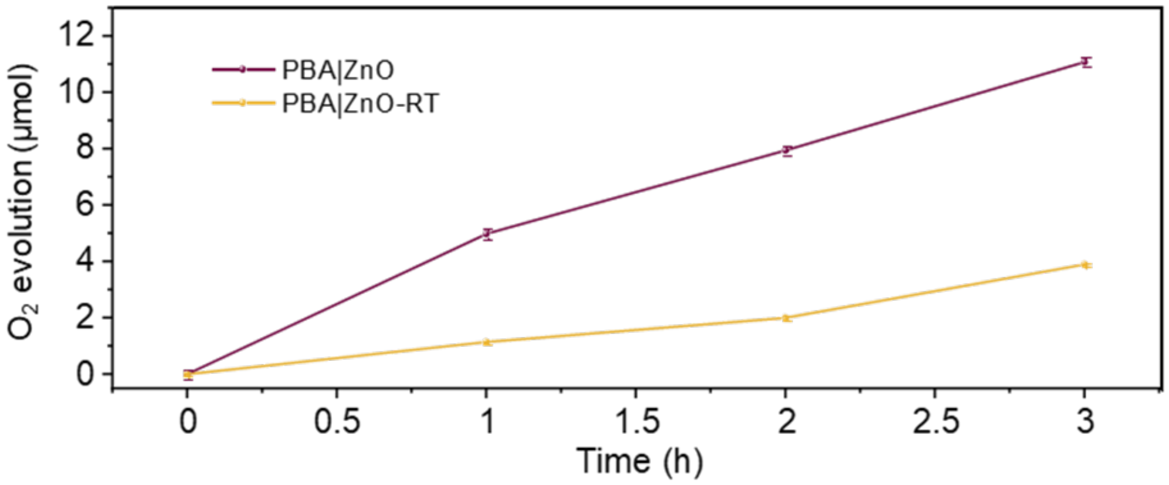

Figure S20. Oxygen evolution was measured hourly during photocatalysis with PBA|ZnO and PBA|ZnO-RT. Each data point was corrected against a blank reference measurement. Initially, at zero-point (in the dark), no O_2 was detected, indicating that the measured O_2 is exclusively a product of light-induced O_2 evolution by the catalyst. Furthermore, the results suggest that the pre-oxidized PBA at the buried PBA|ZnO interface does not contribute to O_2 evolution in the dark (within the detection limit). It becomes active only under light irradiation, where it facilitates interfacial hole transfer and the generation of oxidized PBA (the active catalyst) through intermolecular cooperative interactions, resulting in the superior performance of PBA|ZnO compared to PBA|ZnO-RT.

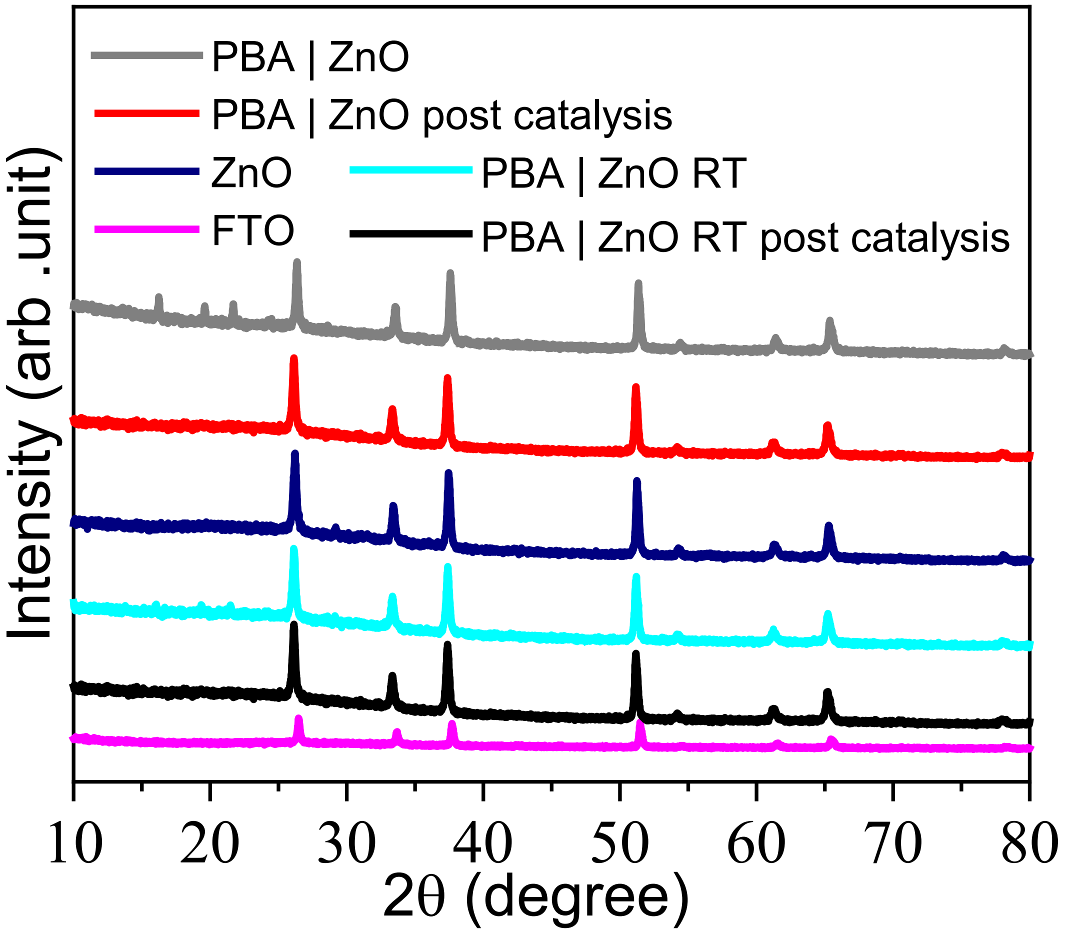

Figure S21. PXRD patterns of PBA|ZnO and PBA|ZnO RT, before and after photocatalytic water oxidation for 3h under solar simulator.

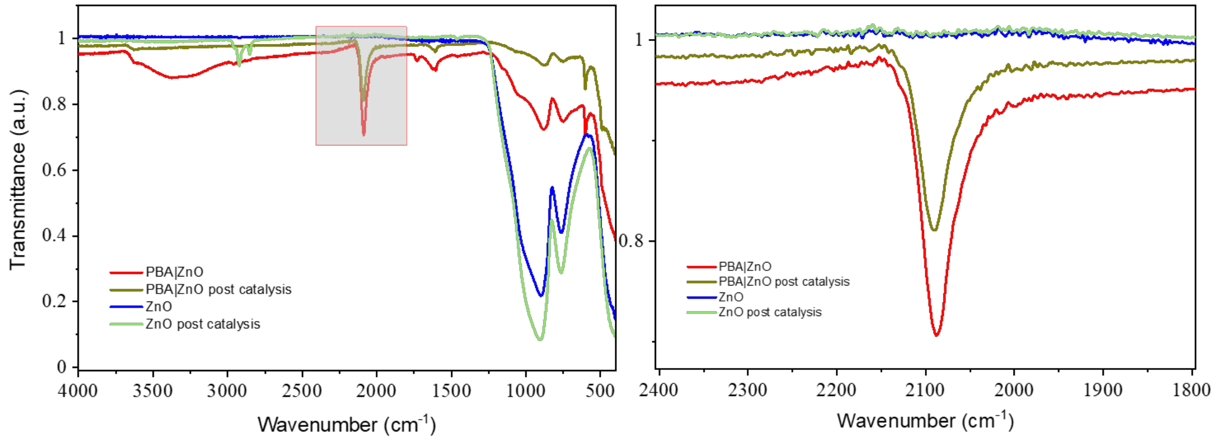

Figure S22. ATR-IR spectrum of ZnO and PBA|ZnO surface before and after photocatalytic water oxidation for 3h under the solar simulator.

The ATR-IR spectra of the pristine and post-catalytic samples are shown in Figure S22. The intense cyanide stretching vibration in PBA-coated samples is observed at 2087 cm^{-1}, which is

assigned to Co^{II}-NC-Fe^{II} coordination mode(bulk composition).[5] A broad band around 3350 cm^{-1} is also observed in PBA|ZnO. This band is assigned to OH stretching mode of the water molecules adsorbed on the surface during the fabrication of PBA|ZnO at 75°C. The intense band ~1000 nm originates from the underlying FTO substrates.

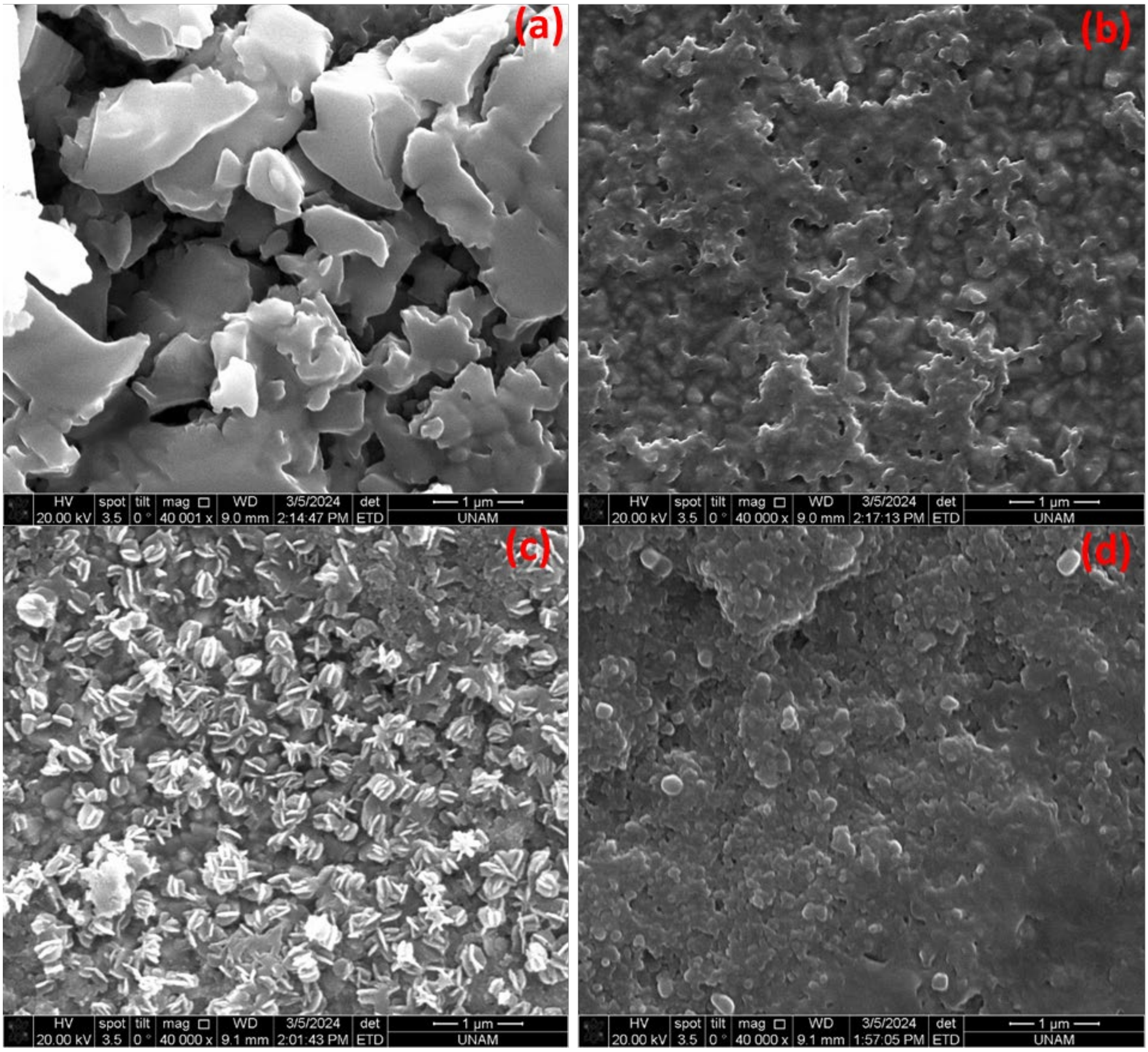

Figure S23. SEM micrograms of ZnO (a) before and (b) after catalysis, of PBA|ZnO (c) before and (d) after and after photocatalytic water oxidation for 3h under the solar simulator.

References

[1] A. A. Ahmad, T. G. Ulusoy Ghobadi, M. Buyuktemiz, E. Ozbay, Y. Dede, F. Karadas, *Inorganic Chemistry* **2022**, *61*, 3931-3941.

[2] R. De, A. Bera, H. Schmidt, C. Neumann, W. Paa, A. Gawlik, A. Turchanin, B. Dietzek-Ivanšić, *ChemPhysChem* **2023**, *24*, e202300203.

[3] M. W. Allen, C. H. Swartz, T. H. Myers, T. D. Veal, C. F. McConville, S. M. Durbin, *Physics Review B* **2010**, *81*, 075211.

[4] E. H. Backus, S. Hosseinpour, C. Ramanan, S. Sun, S. J. Schlegel, M. Zelenka, X. Jia, M. Gebhard, A. Devi, H. I. Wang, *Angewandte Chemie International Edition* **2024**, *63*, e202312123.

[5] R. O. Lezna, R. Romagnoli, N. R. de Tacconi, K. Rajeshwar, *Journal of Physical Chemistry B* **2002**, *106*, 3612-3621.
